# SARS-CoV-2 infection induces the dedifferentiation of multiciliated cells and impairs mucociliary clearance

**DOI:** 10.1038/s41467-021-24521-x

**Published:** 2021-07-16

**Authors:** Rémy Robinot, Mathieu Hubert, Guilherme Dias de Melo, Françoise Lazarini, Timothée Bruel, Nikaïa Smith, Sylvain Levallois, Florence Larrous, Julien Fernandes, Stacy Gellenoncourt, Stéphane Rigaud, Olivier Gorgette, Catherine Thouvenot, Céline Trébeau, Adeline Mallet, Guillaume Duménil, Samy Gobaa, Raphaël Etournay, Pierre-Marie Lledo, Marc Lecuit, Hervé Bourhy, Darragh Duffy, Vincent Michel, Olivier Schwartz, Lisa A. Chakrabarti

**Affiliations:** 1 Virus & Immunity Unit, Department of Virology, Institut Pasteur, Paris, France; 2 UMR 3569 CNRS, Paris, France; 3 Lyssavirus Epidemiology and Neuropathology Unit, Institut Pasteur, Paris, France; 4 Perception and Memory Unit, Institut Pasteur, Paris, France; 5 UMR 3571 CNRS, Paris, France; 6 Translational Immunology Lab, Department of Immunology, Institut Pasteur, Paris, France; 7 Biology of Infection Unit, Institut Pasteur, Paris, France; 8 INSERM U1117, Paris, France; 9 UtechS Photonics BioImaging, C2RT, Institut Pasteur, Paris, France; 10 Image Analysis Hub, C2RT, Institut Pasteur, Paris, France; 11 UtechS Ultrastructural BioImaging UBI, C2RT, Institut Pasteur, Paris, France; 12 Institut de l’Audition, Institut Pasteur, INSERM, Paris, France; 13 Biomaterials and Microfluidics Core Facility, Institut Pasteur, Paris, France; 14 Université de Paris, Necker-Enfants Malades University Hospital, Division of Infectious Diseases and Tropical Medicine, APHP, Institut Imagine, Paris, France; 15 Vaccine Research Institute, Créteil, France

**Keywords:** Cilia, SARS-CoV-2

## Abstract

Understanding how SARS-CoV-2 spreads within the respiratory tract is important to define the parameters controlling the severity of COVID-19. Here we examine the functional and structural consequences of SARS-CoV-2 infection in a reconstructed human bronchial epithelium model. SARS-CoV-2 replication causes a transient decrease in epithelial barrier function and disruption of tight junctions, though viral particle crossing remains limited. Rather, SARS-CoV-2 replication leads to a rapid loss of the ciliary layer, characterized at the ultrastructural level by axoneme loss and misorientation of remaining basal bodies. Downregulation of the master regulator of ciliogenesis Foxj1 occurs prior to extensive cilia loss, implicating this transcription factor in the dedifferentiation of ciliated cells. Motile cilia function is compromised by SARS-CoV-2 infection, as measured in a mucociliary clearance assay. Epithelial defense mechanisms, including basal cell mobilization and interferon-lambda induction, ramp up only after the initiation of cilia damage. Analysis of SARS-CoV-2 infection in Syrian hamsters further demonstrates the loss of motile cilia in vivo. This study identifies cilia damage as a pathogenic mechanism that could facilitate SARS-CoV-2 spread to the deeper lung parenchyma.

## Abstract (web summary)

SARS-CoV-2 infection damages the airways. Here the authors show that SARS-CoV-2 infection induces the rapid loss of airway motile cilia, resulting in altered cilia clearance function. Cilia loss is preceded by reduced expression of the ciliogenesis regulator Foxj1.

## Introduction

The COVID-19 pandemic remains a worldwide public health emergency. The severe acute respiratory syndrome coronavirus 2 (SARS-CoV-2) was first detected in pneumonia patients from Wuhan, China, in December 2019^1–3^ and has now spread to all continents except Antarctica, affecting over 140 million persons and causing over 3 million deaths as of April 2021 (covid19.who.int).

The respiratory syndrome COVID-19 ranges from mild upper respiratory tract infection to bilateral pneumonia with acute respiratory distress syndrome (ARDS) and multiple organ failure^4–6^. Pathological examination indicates that SARS-CoV-2 targets primarily the airways and the lungs^7,8^. Severe cases are characterized by diffuse alveolar damage and formation of hyaline membranes that limit gaseous exchanges. COVID-19 pneumonia is associated with inflammatory infiltrates in the alveolar space and a systemic cytokine storm, suggesting that an exacerbated immune response contributes to damaged lung function^4,9^. Induction of interferons appears limited in the more severe clinical cases, pointing to an imbalance between antiviral and inflammatory cytokine responses^10,11^.

SARS-CoV-2 is genetically closely related to the original SARS-CoV-1 coronavirus, which caused an outbreak of severe acute respiratory syndrome in 2002–2003, affecting about 8000 patients and resulting in 774 reported deaths^12,13^. Both viruses use the angiotensin converting enzyme 2 (ACE2) as a receptor, and the transmembrane serine protease 2 (TMPRSS2) as a viral entry cofactor^2,14^. The pneumonia induced by the two coronaviruses show similar features in severe cases. SARS-CoV-2 induces a mortality rate approximately ten times lower than SARS-CoV-1, but shows much higher effective transmissibility, and thus represents a greater threat to global health^13^. Possible reasons for the high transmissibility of SARS-CoV-2 include an active viral replication in upper airway epithelia at an early stage of infection. The number of SARS-CoV-2 genomic copies in nasopharyngeal swabs is generally high at symptom onset (≥10^6^ viral RNA copies/mL) and persists for about 5 days before declining^15,16^. This is in contrast to the pattern observed for SARS-CoV-1, with viral RNA peaking about 10 days after symptom onset and remaining of moderate magnitude^12^. Therefore, analyzing how SARS-CoV-2 spreads in the airways is relevant to understand its pandemic potential and potentially identify novel mitigation strategies.

The epithelium lining the airways plays a key role in the defense against infections^17^. It comprises goblet cells that secrete a protective mucus able to trap inhaled particles, including microbes. Ciliated cells, which constitute over half of epithelial cells, possess an apical layer of about 200 cilia that beat rhythmically in a coordinated fashion, resulting in a movement of the overlaying mucus layer towards the laryngopharynx, where it is ultimately swallowed^18^. This mechanism of mucociliary clearance prevents the accumulation of particles and mucus within the lungs. The airways basal cells, located close to the epithelial basement membrane, respond to injury by proliferating and differentiating into other epithelial cell types. Studies of autopsy samples from COVID-19 patients and experimental infection of tissue explants have documented SARS-CoV-2 replication predominantly in the upper and lower airway epithelia and in the lung alveoli^19–21^. Infection of reconstructed airway epithelia have shown a preferential targeting of ciliated cells^1,20,22,23^, with damage to these cells documented by a loss of the ciliated layer, the presence of apoptotic cells, and an impairment of epithelial tight junctions^24–26^. Infection of goblet cells appeared less prominent and was documented in some^23–25^ but not all studies^1,20^, with a possible expansion of SARS-CoV-2 tropism in late stage infection^22^. Basal cells were rarely or not infected, and contributed to epithelial repair by differentiating into new ciliated cells^22,25,26^. This process generated a new population of target cells susceptible to SARS-CoV-2 infection, resulting in a cyclic infection pattern in epithelial cultures maintained in the long term^26^. Other human coronaviruses, including SARS-CoV and the common cold coronaviruses HCoV-NL63, -OC43, and -HKU1 were also shown to target ciliated cells^24,27–29^, pointing to the importance of this cell type in coronavirus pathogenesis. The consequences of SARS-CoV-2 infection on ciliated cell differentiation and functions, however, remain to be fully characterized.

To better understand the mechanism of SARS-CoV-2 dissemination in the respiratory tract, we analyzed the ultrastructural and functional changes induced by infection in a reconstructed human bronchial epithelium model. This system enabled the study of SARS-CoV-2 interactions with its primary target cells in a well-differentiated pseudostratified epithelium. We also examined the impact of SARS-CoV-2 infection on the airway mucosa in vivo, using the physiologically relevant Syrian hamster model.

## Results

### SARS-CoV-2 actively replicates in a reconstructed human bronchial epithelium model

**Fig. 1:**
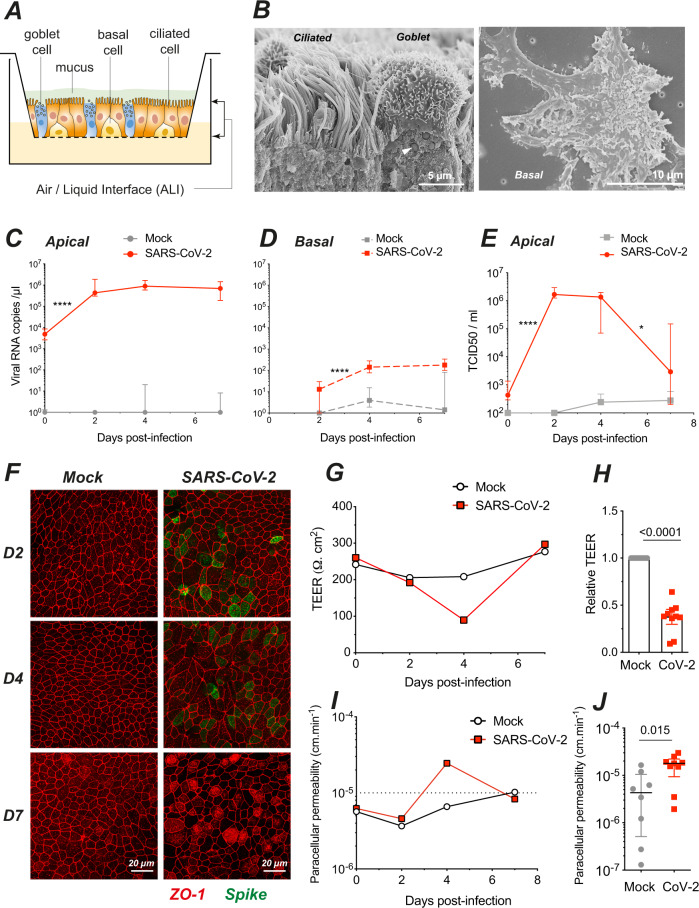
SARS-Cov-2 infection transiently impairs epithelial barrier function in a reconstructed human bronchial epithelium. **A** Schematic view of an Air/Liquid Interface (ALI) culture of a reconstructed human bronchial epithelium comprising three cell types: ciliated, goblet, and basal cells. **B** SEM imaging of the three cell types, with a ciliated and a goblet cell (left panel; arrowhead: mucus granule) and a basal cell (right panel). **C, D** SARS-CoV-2 viral load quantification in apical (**C**) and basal (**D**) culture supernatants by RT-qPCR (**C**: *n* = 7 independent experiments; **D**: *n* = 5; **C, D**: 1–7 replicates per experiment). **E** Infectious SARS-CoV-2 particles production quantified by TCID_50_ on Vero cells (*n* = 2 independent experiments, with 1–8 replicates per experiment). **C–E** Medians ± IQR were compared by Mann–Whitney tests; statistically significant differences between infected samples compared at adjacent days are reported (*P < 0.05, **P < 0.01, ***P < 0.001, ****P < 0.0001). Comparisons between mock and infected samples on the same day were all significant (*P* < 0.001), except in **D** and **E** at 7 dpi. **F** Visualization of tight junctions (ZO-1, red) and infection (SARS-CoV-2 spike, green) by immunofluorescence at 2, 4, and 7 dpi in *n* = 1 representative experiment out of 2. **G–J** Assessment of epithelium barrier function: Trans-Epithelial Electric Resistance (TEER) (**G, H**) and paracellular permeability for dextran-FITC (**I, J**). Panels **G, I** show representative experiments, while panels **H, J** show pooled results at 4 dpi (*n* = 4 independent experiments, with 1–4 replicates per experiment). Median ± IQR values were compared with Mann–Whitney tests.

SARS-CoV-2 infection was studied in the MucilAir^TM^ model, consisting of primary human bronchial epithelial cells grown over a porous membrane and differentiated at the air/liquid interface (ALI) for over 4 weeks (Fig. 1A). We first verified by scanning electron microscopy (SEM) that the bronchial epithelial cells were differentiated into a pseudostratified epithelium comprising three main cell types (Fig. 1B): ciliated cells harboring a dense layer of 5–10 µm long motile cilia, goblet cells enriched in mucus-containing vesicles (arrowhead), and basal cells spread flat on the insert membrane.

The reconstructed epithelia were infected with a viral suspension containing 10^6^ SARS-CoV-2 plaque-forming units (pfu), corresponding to a multiplicity of infection close to 1. The viral suspension was deposited on the apical side for 4 h, restored to ALI conditions, and monitored for infection for 7 days. We observed a rapid increase of extracellular viral RNA in apical culture supernatants (*P* < 0.0001), with concentrations reaching up to 10^6^ viral RNA copies/µL at 2 days post-infection (dpi), followed by stable or slowly decreasing viral RNA levels until 7 dpi (Fig. 1C). In contrast, minimal concentrations of viral RNA were detected in the basolateral compartment (Fig. 1D), indicating that SARS-CoV-2 particles were predominantly released from the apical side of the epithelium. Infectious viral particle production in apical supernatants initially tracked with viral RNA production, with a rapid increase at 2 dpi (*P* < 0.0001) to reach a mean of 1.8 × 10^6^ TCID_50_/mL, followed by a plateau at 4 dpi (Fig. 1E). A decrease to 6.1 × 10^4^ TCID50/mL was then observed at 7 dpi (*P* < 0.05), suggesting a partial containment of infectious virus production. The persistence of high levels of viral RNA at 7 dpi in spite of the decrease in infectious titer may have resulted from a release of viral RNA from lyzed infected cells. Immunofluorescence analysis of the reconstructed epithelia revealed a patchy distribution of infected cells expressing the SARS-CoV-2 spike protein at 2 and 4 dpi, and minimal persistence of productively infected cells at 7 dpi, consistent with partial viral containment after one week of infection (Fig. 1F).

### SARS-CoV-2 infection transiently impairs epithelial barrier function

SARS-CoV-2 productive infection locally altered the distribution of zonula occludens protein-1 (ZO-1), which associates to tight junctions. The characteristic ZO-1 staining pattern at cell boundaries remained intact in mock-infected epithelia (Fig. 1F, left), but appeared disrupted in areas with viral antigen expression (Fig. 1F, right), suggesting a possible impairment of epithelial barrier integrity. Image analysis confirmed that the average number of neighbors per cell at 4 dpi decreased from 5.93 to 5.72 (*P* < 0.001), a value characteristic of actively remodeling epithelia^30,31^ (Supplementary Fig. 1A-H, J). In addition, infection induced changes in the average area of ZO-1-delimited cells, which significantly increased at 2 and 4 dpi, but decreased at 7 dpi as compared to uninfected samples (Supplementary Fig. 1A-I), supporting a dynamic remodeling of the epithelium. The epithelial packing geometry returned to a more regular pattern at 7 dpi, with a higher proportion of hexagonal cells than at 4 dpi, pointing to the restoration of the tight junction network.

To test barrier function, we measured the trans-epithelial electrical resistance (TEER) between electrodes placed in the apical and basal compartments of reconstructed bronchial epithelia (Fig. 1G). As expected, TEER proved relatively stable in mock-treated cultures, with values remaining above 200 Ω.cm^2^. In SARS-CoV-2-infected cultures, there was a transient but highly significant 3.3× decrease in relative TEER at 4 dpi (Fig. 1H; *P* < 0.0001). Apical-to-basolateral transport of FITC-coupled dextran showed an inverse relation to TEER, with a transient 2.5× increase in paracellular permeability at 4 dpi (Fig. 1I, J; *P* = 0.015). Both TEER and permeability values returned to baseline levels at 7 dpi, demonstrating epithelial regeneration after SARS-CoV-2 infection.

Measurement of cell death by the release of lactate dehydrogenase (LDH) in apical supernatants showed an increase at 4 dpi in infected epithelia (Supplementary Fig. 2A), confirming that SARS-CoV-2 exerted a transient cytopathic effect on epithelial cells. Dying cells extruded from the apical side were observed at the surface of infected epithelia, although their numbers remained limited (Supplementary Fig. 2B). Extruded cells had a rounded shape and sometimes carried multiple viral particles at their surface (Supplementary Fig. 2C), suggesting virally induced cell death. Taken together, these findings showed that SARS-CoV-2 caused a transient loss of epithelial barrier function, due to cytopathic effect and a perturbation of the tight junction network. However, SARS-CoV-2 infection did not cause a general disruption of the epithelial layer and was compatible with rapid epithelial regeneration.

### SARS-CoV-2 infection damages the ciliary layer

**Fig. 2:**
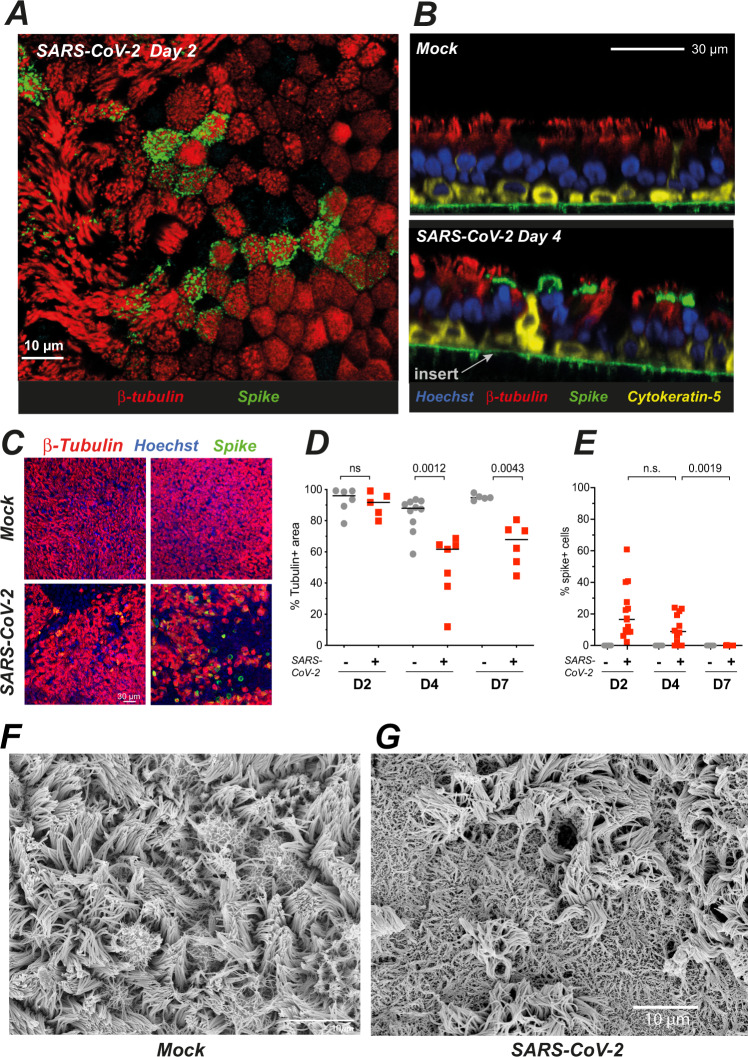
SARS-CoV-2 preferentially targets ciliated cells and damages the ciliary layer. **A, B** Confocal imaging of a SARS-CoV-2-infected epithelium at 2 dpi (**A**, top section) and of control (Mock) and infected epithelia at 4 dpi (**B**, orthogonal sections). Ciliated cells are labeled for β-tubulin IV (red), basal cells for cytokeratin-5 (yellow), nuclei for DNA (Hoechst, blue), and infected cells for the SARS-CoV-2 spike (green). The green line in 2B is due to the autofluorescence of the insert porous membrane. **C, D** Representative images of the β-tubulin layer at 4 dpi (**C**) and corresponding image analysis (**D**) (*n* = 3 independent experiments with 1–4 replicates for D4; *n* = 2 independent experiments with 2–3 replicates for D2 and D7; Mann–Whitney test). **E** Percentage % of spike+ cells, measured as (number of spike+ cell/number of nuclei) × 100 (*n* = 5 independent experiments with 1–5 replicates for D2 and D4; *n* = 3 independent experiments with 3 replicates for D7; Mann–Whitney test). **F, G** SEM imaging of mock-infected (**E**) and SARS-CoV-2-infected (**F**) reconstructed epithelia at 4 dpi.

The nature of infected cells was characterized by immunofluorescence confocal imaging. At 2 dpi, the majority of cells expressing the SARS-CoV-2 spike antigen at their surface (spike+ cells) co-expressed the cilia marker β-tubulin IV, confirming that ciliated cells represented the main viral targets in this model (Fig. 2A). At 4 dpi, the majority of spike+ cells still expressed β-tubulin IV, although we noted the presence of infected cells with weak or absent β-tubulin IV staining (Fig. 2B). Basal cells expressing the cytokeratin-5 marker did not appear infected, with the exception of rare cells that had lost their basal localization (Supplementary Fig. 3A). Of note, some spike-negative basal cells changed morphology and appeared raised through the pseudostratified epithelium in infected samples, suggesting an epithelial response to virally induced damage (Fig. 2B). We did not detect infected cells expressing the goblet cell marker MUC5AC (Supplementary Fig. 3B). However, further analyses by scanning electron microscopy (SEM) documented viral budding from cells with multiple secretory pores that may represent rare infected goblet cells (Supplementary Fig. 3C). Interestingly, viral production was also documented in cells with a transitional phenotype characterized by the presence of both motile cilia and abundant secretory vesicles (Supplementary Fig. 3D). Taken together, these results showed a preferential tropism of SARS-CoV-2 for ciliated epithelial cells, with occasional infection of transitional and secretory cells.

As we had observed infected cells with weak β-tubulin IV staining, we asked whether SARS-CoV-2 infection could perturb the layer of motile cilia present at the apical side of ciliated cells. To this goal, we quantified the area occupied by β-tubulin IV staining (tubulin + area) on projections of confocal images obtained sequentially in the course of infection (Fig. 2C, D). The tubulin+ area remained unchanged at 2 dpi, but decreased at 4 dpi (median values: 87.9% in mock, 61.7% in infected; *P* = 0.0012), and showed only limited recovery at 7 dpi (94.8% in mock vs 67.9% in infected, *P* = 0.0043). In contrast, productively infected cells detected by spike labeling became undetectable at 7 dpi (Fig. 2E). SEM imaging confirmed a marked cilia loss at 4 dpi and showed that deciliated areas were not devoid of cells, but rather occupied by cells covered by flattened microvilli (Fig. 2F, G).

**Fig. 3:**
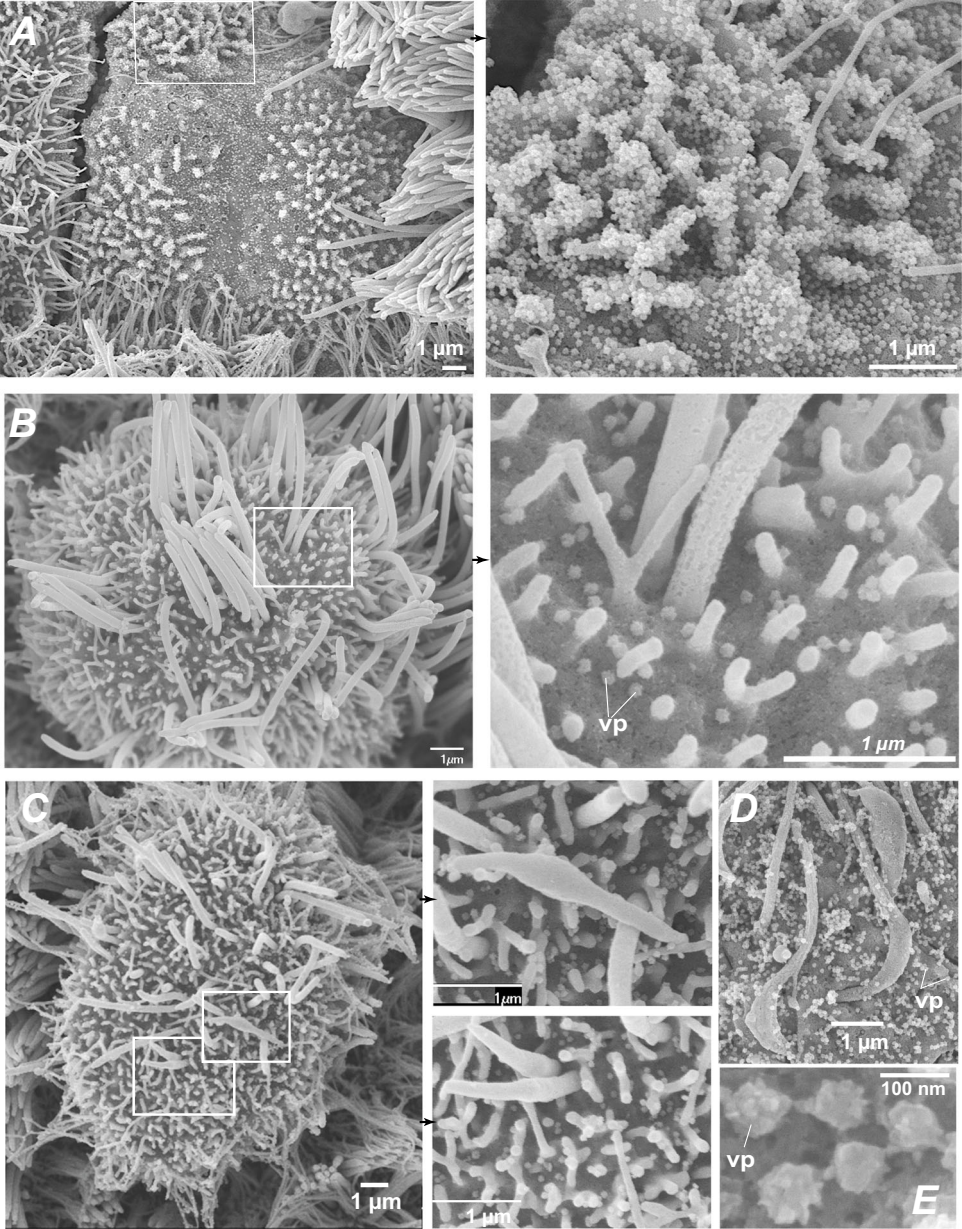
SARS-CoV-2 infection causes an early loss of motile cilia. **A** Scanning electron microscopy (SEM) image of a massively infected cell at 2 dpi (left) with a lack of cilia and an accumulation of viral particles at the surface of membrane ruffles (enlarged in right panel). **B** SEM image of an infected cell at 2 dpi with few remaining cilia (left) and scattered viral particles (vp) at the plasma membrane (right). **C, D** SEM images of infected cells at 2 dpi showing cilia abnormalities, including shortened misshapen cilia (**D** left, enlarged in middle panels) and crescent-shaped proximal axonemes (**E**). **E** SEM image of pleiomorphic SARS-CoV-2 viral particles.

The formation of deciliated areas could occur via the loss of motile cilia at the surface of infected cells, or via the replacement of dead ciliated cells by cells involved in epithelial regeneration. To distinguish between these nonexclusive possibilities, we analyzed the distribution of cilia at the early stage of infection, prior to the occurrence of measurable cell death. A set of ≥70 cells was analyzed on confocal images obtained at 2 dpi in each of three categories: ciliated cells from mock-infected epithelia (mock), productively infected ciliated cells (spike+) and bystander uninfected ciliated cells (spike−) from SARS-CoV-2 exposed epithelia. For each cell, the averaged intensity profile of β-tubulin IV staining was measured along the depth axis (Supplementary Fig. 4A, B). There was a bimodal distribution of β-tubulin in mock cells, with a distal peak corresponding to cilia and a proximal peak located just below the plasma membrane. This proximal peak corresponded to the area where basal bodies anchor cilia into the cytoplasm, but may also have included microtubules present in the apical cytoskeleton. Examination of average profiles for each category suggested a specific decrease of the distal β-tubulin peak in spike+ cells. This was confirmed by an analysis of the distal to proximal peak intensity ratio, which showed a significant decrease in spike+ cells, as compared to spike− and mock cells (Supplementary Fig. 4C). Thus, the density of cilia decreased at 2 dpi, supporting an early loss of cilia in infected cells. SEM imaging confirmed the presence of productively infected cells with only few remaining cilia on their apical surface (Fig. 3), as opposed to the packed ciliary layer characteristic of intact ciliated cells (Fig. 1B). Some infected cells showed a lack of cilia and a massive accumulation of virions at the cell surface and on membrane ruffles (Fig. 3A), indicative of highly productive SARS-CoV-2 infection. Of note, viral particles were rarely observed along the length of ciliary sheaths (Fig. 3B). This observation was consistent with the distribution of spike staining, which formed a narrow band above the plasma membrane, but did not overlay the distal β-tubulin peak (Supplementary Fig. 4A, B, spike+ cell). In addition, spike and β-tubulin labeling showed minimal colocalization, as measured by Mander correlation coefficients (Supplementary Fig. 4D-E). Thus, viral particle release or accumulation did not take place in the cilium structure, suggesting that cilia destruction occurred through an indirect mechanism.

### SARS-CoV-2 infection induces ultrastructurally abnormal cilia

**Fig. 4:**
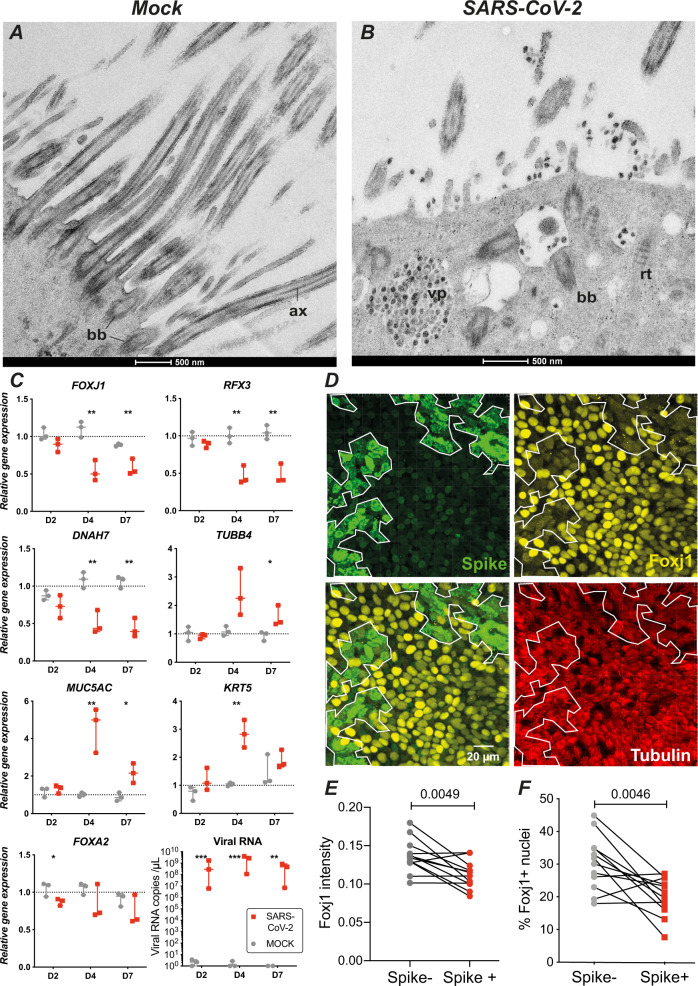
SARS-CoV-2 infection perturbs ciliogenesis and induces Foxj1 downregulation. **A** TEM image of a mock-infected ciliated cell with elongated axonemes (ax) and membrane proximal basal bodies (bb). **B** TEM image of an infected ciliated cells at 4 dpi, with viral particle (vp) accumulation, mislocalized basal bodies, and isolated rootlets (rt). lp large particle. **C** Quantitation of transcripts expressed in mock- and SARS-CoV-2-infected epithelia (median ± range). The expression of 8 genes was measured by qPCR in *n* = 3 biological replicates from different donors and compared by *t*-tests with false discovery rate (FDR) correction for multiple comparisons (*P < 0.05, **P < 0.01, ***P < 0.001). **D** Immunofluorescence analysis of epithelia at 2 dpi, after labeling for the viral spike (green), Foxj1 (yellow), and β-tubulin IV (red). The limit of the Spike+ area is reported on each panel (white line). **E, F** The Foxj1 mean fluorescence intensity in 3D-segmented nuclei (**E**) and the percentage of Foxj1+ nuclei (**F**) were compared in Spike− and Spike+ areas for *n* = 4 donors with 3 replicates per donor, using a paired *t*-test.

SEM imaging performed at higher magnification revealed ultrastructural abnormalities in infected ciliated cells. Cilia were often shortened and misshapen (Fig. 3C) and sometimes showed crescent-shaped juxta-membrane regions (Fig. 3D). Viral particles present at the membrane were not symmetrical but rather pleiomorphic (Fig. 3E), consistent with studies suggesting that spike trimers adopt various orientations at the virion surface^32^. Transmission electron microcopy (TEM) was then used to examine the internal organization of cilia. Mock-infected epithelia revealed a typical “9 + 2” cilium structure, with peripheral microtubule doublets surrounding a central microtubule pair (Supplementary Fig. 5 A). These microtubules constituted elongated axonemes that emerged from basal bodies aligned perpendicular to the plasma membrane (Fig. 4A). The basal bodies were themselves anchored into the cytoplasm through striated rootlets (Supplementary Fig. 5B). A striking disorganization of cilia structure was observed in SARS-CoV-2-infected cells, with fewer axonemes, and misoriented basal bodies that lined large vesicles, which themselves often contained viral-like particles (Figs. 4B and S5C, D). Isolated rootlets were also detected, suggesting a dissociation of ciliary components (Fig. 4B). The vesicles contained packed viral-like particles, but also larger particles that may have derived from engulfed basal bodies (Figs. 4B and S5C). Rarely, shortened cilia were also detected inside vesicles (Supplementary Fig. 5E). Taken together, SARS-CoV-2 infection had a major impact on ciliary structure, by inducing misshapen cilia, axoneme loss, and accumulation of mislocalized basal bodies.

### SARS-CoV-2 infection inhibits the expression of the ciliogenesis regulator Foxj1

We next analyzed the expression of key genes involved in regulating ciliogenesis. Quantitative RT-PCR analysis showed a progressive decrease in transcripts encoding the ciliary component *DNAH7* and ciliogenesis regulators *FOXJ1* and *RFX3* upon SARS-CoV-2 infection (Fig. 4C), consistent with a progressive dedifferentiation or loss of multiciliated cells. In contrast, an increase in transcripts expressed in goblet cells (*MUC5AC*) and in basal cells (cytokeratin-5 *KRT5*) suggested compensatory changes involved in epithelial repair. *TUBB4*, coding for β-tubulin IV, showed an increased expression starting from 4 dpi, possibly to compensate for the loss of this β-tubulin isoform observed at the protein level (Fig. 2D). The epithelial gene *FOXA2* showed only a minor decrease at 2 dpi, compatible with an overall preservation of the epithelial layer upon infection.

We had noted an early thinning of the ciliary layer in infected cells at 2 dpi (Supplementary Fig. 4A, B), while we did not detect a significant decrease in transcripts of the master regulator of ciliogenesis *FOXJ1* at this timepoint. As *FOXJ1* expression was recently shown to be regulated at a post-transcriptional level^33^, we analyzed the expression of the Foxj1 protein at the early stage of infection. Immunofluorescence analysis of revealed a decreased expression of the Foxj1 protein at 2 dpi, specifically in infected areas that expressed the spike antigen (example in Fig. 4D). Low Foxj1 expression was observed in spike+ areas that still showed high levels of β-tubulin IV, suggesting that loss Foxj1 preceded that of cilia (Fig. 4D, bottom right panel). Quantitative image analysis in 3D-segmented nuclei confirmed a significant decrease of Foxj1 fluorescence intensity in spike+ areas of infected epithelia at 2 dpi (Fig. 4E). Computation of the percentage of Foxj1+ nuclei further supported an early loss of Foxj1 in infected areas (Fig. 4F). Therefore SARS-CoV-2 infection induced a decrease in Foxj1 protein expression at 2 dpi, while *FOXJ1* transcript were not yet impacted at this stage, pointing to a post-transcriptional negative regulation. These findings documented an early perturbation of ciliogenesis regulation upon SARS-CoV-2 infection, which could help explain the dedifferentiation of multiciliated cells observed at the ultrastructural level.

### SARS-CoV-2 infection impairs mucociliary clearance

**Fig. 5:**
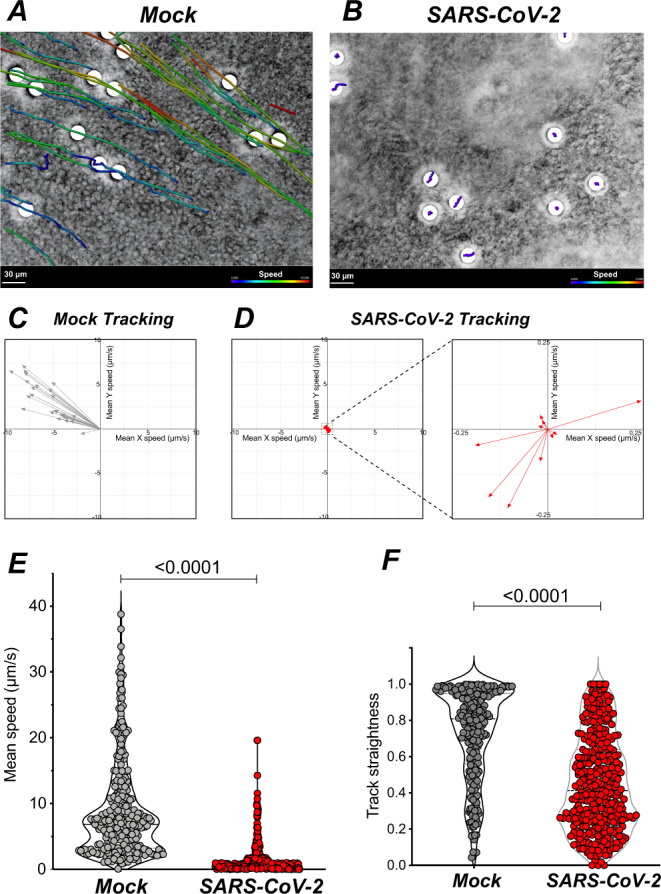
SARS-CoV-2 infection impairs mucociliary clearance. **A, B** Representative examples of bead tracking on mock (**A**) and infected (**B**) epithelia at 7 dpi. Bead speed is color-coded in each track. **C, D** Vector representation of tracks from **A** and **B** showing bead speed and direction. A low bead speed is observed in infected epithelia (**D**, left), with an enlarged view showing non-uniform directions (**D**, right). **E, F** Bead mean speed (**E**) and track straightness (**F**) measured in mock and infected epithelia at 7 dpi (*n* = 3 independent experiments, with 68–118 beads tracked per sample; Mann–Whitney tests).

We next examined the consequences of SARS-CoV-2 infection on ciliary function. To this goal, we deposited low density 30 µm-sized polystyrene microbeads onto the apical surface of mock-treated or infected epithelia and tracked their movement in real time. These experiments were performed at 7 dpi, to allow sufficient reconstitution of the mucus layer after the infection step. We observed that beads deposited on mock-treated epithelia moved generally in the same direction, consistent with coordinated beating of the underlying cilia (Fig. 5A, C, and Supplementary Movie 1). In contrast, beads deposited on infected epithelia were mostly immobile or showed randomly-oriented limited movements, indicating an impairment of the mucociliary clearance function (Fig. 5B, D, and Supplementary Movie 2). Quantitation of velocities confirmed a highly significant decrease in bead clearance upon SARS-CoV-2 infection (mean: 8.9 µm/s in mock vs 1.5 µm/s in infected epithelia; *P* < 0.0001) (Fig. 5E). The straightness of tracks also decreased in infected epithelia (Fig. 5F; *P* < 0.0001), suggesting a perturbation in the coordination of cilia movements. Thus, cilia alterations induced by SARS-CoV-2 infection were associated to a marked impairment in mucociliary transport.

### SARS-CoV-2 infection triggers epithelial defense mechanisms

**Fig. 6:**
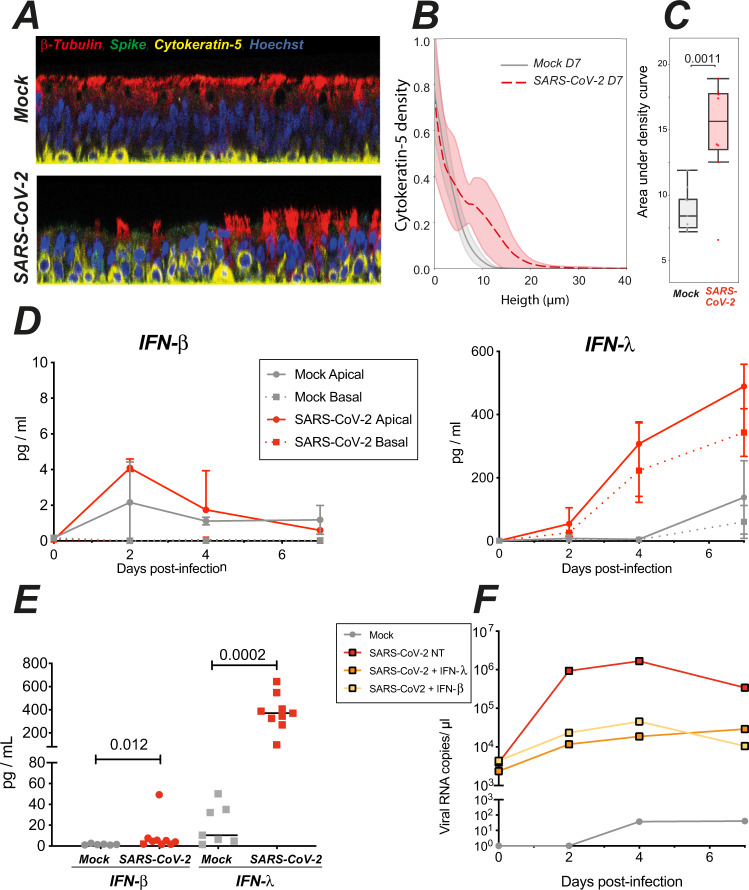
SARS-CoV-2 infection triggers epithelial defense mechanisms: basal cell mobilization and interferon induction. **A** Representative confocal images from mock and infected epithelia obtained at 7 dpi and used for quantification of basal cell (cytokeratin-5+, yellow) position along the *Z*-axis (same color code as in 4B). **B, C** Quantification of basal cell mobilization by analysis of the cytokeratin-5 density profile along the *Z*-axis. **B** The mean density ± SD of 8 (SARS-CoV-2) or 9 (Mock) images from *n* = 3 independent experiments is shown. **C** Comparison of areas under the curves shown in **B** with an unpaired *t*-test. Box plots show median and interquartile range (box) and 1.5× the interquartile range (whiskers), with all points shown. **D** Kinetics of IFN-β (left) and IFN-λ (right) production in apical and basal supernatants of mock and infected epithelia (median ± IQR, *n* = 2 independent experiments, 1–4 replicates per experiment). **E** Quantification of interferon production in apical supernatants at 4 dpi (medians for *n* = 4 independent experiments, 1–4 replicates per experiment). Statistical differences between infected and mock samples were compared by the Mann–Whitney test. **F** Effect of interferon pretreatment on SARS-CoV-2 replication in reconstructed epithelia. Viral RNA copies were quantified in apical supernatants by qRT-PCR (*n* = 1 experiment).

Mucociliary transport was altered at 7 dpi, at a time when barrier integrity was already restored (Fig. 1). To better apprehend the process of epithelial regeneration, we analyzed the localization and morphology of basal cells at this timepoint. Confocal images showed that basal cells expressing cytokeratin-5 were typically flattened on the basement membrane of mock-treated epithelia, while they appeared raised through the thickness of the pseudostratified epithelium in infected samples (Fig. 6A). To quantify this phenomenon, we first generated elevation maps of the inserts that support the cultures, and used these to correct for local insert deformations (Supplementary Fig. 6A). This approach enabled to precisely quantify the mean height of basal cells in the epithelia, which proved significantly higher in infected than noninfected samples (Fig. 6B, C). SEM imaging confirmed that basal cells adopted a more rounded morphology in infected epithelia (Supplementary Fig. 6B, C). Thus, basal cells were mobilized at 7 dpi, which may contribute to the restoration of barrier integrity. Of note, we use herein the term mobilization to describe the relocalization of basal cells through the pseudostratified epithelium, which in this particular case does not imply a migration from another tissue. Basal cells had not yet differentiated into ciliary cells at 7 dpi, as attested by the persistence of deciliated areas (Fig. 2D) and the impairment in mucociliary clearance (Fig. 5E). In a long-term infection experiment, viral infectious titers showed signs of cyclic fluctuations with peaks at 4, 9, and 18 dpi (Supplementary Fig. 7 A, B). This observation suggests that ciliated cells could differentiate after the first wave of infection and provide new target cells at later time points, consistent with the findings of Hao et al.^26^. Interestingly, the relative TEER values fluctuated inversely to the infectious titers (Supplementary Fig. 7C, D), suggesting alternating phases of epithelial damage and repair.

Epithelial interferon production provides another key defense mechanism, that can occur in the absence of immune cell infiltration, and thus represents one of the earliest antiviral response to respiratory viruses. We monitored the kinetics of type I (IFN-α, IFN-β) and type III (IFN-λ) interferon production in the supernatants of reconstructed epithelia. IFN-α2 remained undetectable, using a high-sensitivity SIMOA assay with a limit of detection (LOD) of 2 fg/mL^11^. IFN-β was minimally induced upon SARS-CoV-2 infection, with peak values of 4.2 ± 0.4 pg/mL at 2 dpi in apical supernatants, and levels below the LOD (<1.7 pg/mL) in basal supernatants (Fig. 6D, left). In contrast, IFN-λ induction showed a different kinetics, with limited induction in apical supernatants at 2 dpi, but persistent increase at 4 and 7 dpi, to reach relatively high concentrations in both the apical (501.3 ± 81.8 pg/mL) and basal supernatants (343 ± 106 pg/mL) (Fig. 6D, right). Both IFN-β and IFN-λ production showed a degree of intersample variability, but were significantly induced as compared to mock-treated samples at 4 dpi (Fig. 6E). It remained striking, however, that viral replication already peaked at 2 dpi (Fig. 1E), while interferon production was minimal at this stage. Pretreatment of the reconstructed epithelia with exogenous IFN-β or IFN-λ prior to SARS-CoV-2 infection decreased viral RNA levels by 2 logs (Fig. 6F), pointing to the importance of the timing of IFN induction to achieve viral containment. Taken together, these findings documented the induction of epithelial defense mechanisms following SARS-CoV-2 infection, including basal cell mobilization and type III interferon induction. However, the kinetics of these responses appeared too slow in this model to prevent viral replication and functional impairment.

### SARS-CoV-2 infection damages the ciliary layer in the respiratory tract of Syrian hamsters

**Fig. 7:**
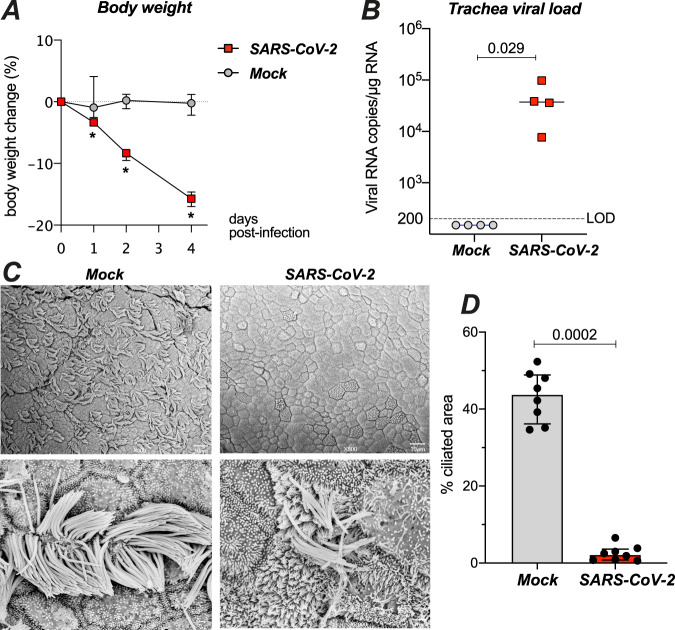
SARS-CoV-2 damages the ciliary layer in the respiratory tract of Syrian golden hamsters. **A** Effect of intranasal SARS-CoV-2 infection on hamster body weight (*n* = 4 per group; median ± IQR). Differences between groups were measured by a Mann–Whitney test (*P < 0.05). **B** SARS-CoV-2 viral load measured at 4 dpi in hamster trachea by RT-qPCR (*n* = 4 per group; medians, Mann–Whitney test). LOD limit of detection. **C** SEM images of tracheal epithelia from mock (left panels) and SARS-CoV-2-infected (right panels) hamsters at 4 dpi. Enlarged views of ciliated cell are shown in bottom panels. **D** Quantification of ciliated area in hamster tracheas imaged by SEM (SARS2: *n* = 3, MOCK: *n* = 2; 2–4 images per sample). Differences between medians ± IQR were measured by a Mann–Whitney test.

**Fig. 8:**
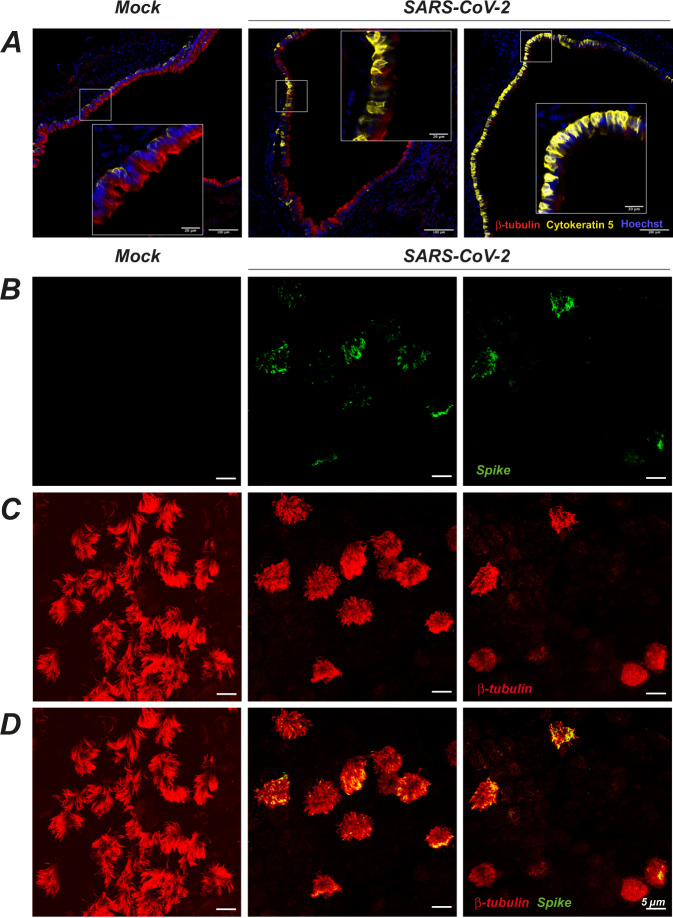
SARS-CoV-2 infection targets ciliated cells in the trachea of infected Syrian golden hamsters. **A** Immunofluorescence imaging of hamster tracheas at 4 dpi. Ciliated cells are labeled with β-tubulin IV (red), basal cells by cytokeratin-5 (yellow), and nuclei by Hoechst (blue). Representative images are shown for a mock-infected animal (left, *n* = 3), and SARS-CoV-2-infected animals who received a low dose (middle, *n* = 2) or a high dose (right, *n* = 2) of virus. **B–D** Whole-mount immunofluorescence imaging of the tracheal epithelium of mock-infected (left) or SARS-CoV-2-infected (middle and right) hamsters at 4 dpi. Labeling for the SARS-CoV-2 spike (**B**, green), the ciliated cell marker β-tubulin IV (**C**, red), or both markers (**D**) are shown. The tracheal epithelium obtained in the infected animal show various degrees of cilia destruction (middle, right) depending on the area.

We next asked whether the ciliated airway epithelium is impacted by SARS-CoV-2 infection in vivo. We chose the golden Syrian hamster (*Mesocricetus auratus*) model, as this species is naturally susceptible to SARS-CoV-2 and shows lung lesions upon infection^34,35^. Syrian hamsters were infected with 6 × 10^4^ pfu of SARS-CoV-2 via the intranasal route. The infected hamsters showed rapid body weight loss as compared to mock-infected controls (*n* = 4 in each group; Fig. 7A, *P* < 0.05). The animals were euthanized at 4 dpi, at a time when the infected group showed a high viral load in the trachea (Fig. 7B). SEM imaging showed that cilia occupied almost half of the epithelial surface in the trachea of control animals (Fig. 7C, left). A marked cilia loss occurred in the trachea of infected animals (Fig. 7C, right), as confirmed by image analysis (median of ciliated area: 2.2% in SARS-CoV-2+ vs 43.8% in Mock, *P* = 0.0002) (Fig. 7D). Immunofluorescence labeling of trachea sections confirmed a partial to complete loss of the ciliated layer at 4 dpi, with a mobilization of basal cells towards the luminal side of the epithelium (Fig. 8A). The SARS-CoV-2 spike antigen could be detected in a few remaining ciliated cells (Fig. 8B-D). Therefore, SARS-CoV-2-infected and damaged the epithelial ciliary layer in a physiologically relevant animal model.

## Discussion

We report here that SARS-CoV-2 preferentially replicates in multiciliated cells and induces their dedifferentiation in a reconstructed human bronchial epithelium model. The resulting loss of motile cilia is associated to an impairment of mucociliary clearance function. We also demonstrate, in the SARS-CoV-2 susceptible hamster model, a loss of motile cilia in the trachea of infected animals. Thus, SARS-CoV-2 specifically damaged airway motile cilia, both in vivo and in vitro. Our findings suggest that cilia loss may also play a role in COVID-19 pathogenesis, as a localized clearance impairment at the site of SARS-CoV-2 replication could facilitate viral spread within the airways. Decreased cilia movement could slow the transport of released virions towards the pharynx and facilitate viral access to deeper regions of the bronchial tree. This process could self-perpetuate, with cycles of localized cilia destruction facilitating SARS-CoV-2 progression towards increasingly more distal regions, until the virus reaches the alveoli and triggers pneumocyte damage.

We did not observe major discontinuities in the tracheal epithelium of infected hamsters. Continuity of the epithelial layer was not disrupted in the in vitro model either, as confirmed by the minimal release of viral particles in the basal compartment. A degree of cytotoxicity was measured by LDH release, but the observation of extruded dead cells remained occasional. Epithelial barrier function was impaired at day 4, as documented by decreased trans-epithelial resistance, increased permeability, and altered distribution of the tight junction protein ZO-1. However, this functional impairment remained transient, with signs of epithelial regeneration such as basal cell mobilization observed at day 7. Our results indicate that SARS-CoV-2 replication has a drastic effect on the ciliary layer while exerting a moderate effect on epithelial barrier integrity. This is in contrast to other respiratory viruses such as influenza A virus (IAV) and enterovirus EV-D68, which induces widespread epithelial cell death associated to a loss of epithelial barrier function^36^. In spite of these differences, it is striking that a majority of respiratory viruses target ciliated cells and use various strategies that all converge in limiting mucociliary clearance. Certain rhinoviruses perturb the synchronicity of ciliary beating, which is sufficient to impair the movement of the overlaying mucus layer^36^. Other viruses including SARS-CoV-2 (this study) but also respiratory syncytial virus (RSV), mouse parainfluenza virus, and influenza B virus can induce motile cilia loss by altering the differentiation of ciliated cells^37,38^. Viruses may also induce a rapid apoptosis of ciliated cells, as seen in IAV infection^39^, or accelerate the shedding of infected ciliated cells from the epithelium, as documented for RSV^40^. Whether the dysfunction or loss of infected ciliated cells systematically promotes the dissemination of viral particles through local mucociliary clearance impairment remains to be demonstrated. The shedding or death of infected ciliated cells may in some cases rather be viewed as an epithelial defense mechanism, through the rapid removal of a viral source. On the other hand, experimental impairment of ciliary movements through chemical treatment was shown to increase IAV infection in vitro^41^, providing evidence that decreasing mucociliary clearance can facilitate viral spread. Decreased clearance leading to mucus accumulation in the airways may also induce coughing and thereby facilitate viral transmission.

The role of mucociliary clearance in limiting respiratory bacterial infections is also well documented^42^, and supported by the recurrence of severe pulmonary infections in patients with inborn errors in genes essential to motile cilia function^43^. The percentage of COVID-19 patients presenting with a bacterial or fungal respiratory co-infection at hospital admission remains moderate^44,45^. However, the occurrence of secondary infections increases in critically-ill COVID-19 patients, in spite of common broad-spectrum antibiotic prophylaxis. One fourth to one third of severe COVID-19 patients experience secondary bacterial or fungal superinfections^46–48^. Secondary infections are associated with worse outcomes^45,46^, emphasizing the possible effect of impaired mucociliary clearance on the risk of severe COVID.

Ultrastructural analysis by SEM revealed a loss of motile cilia in productively infected human bronchial cells. Misshapen and shortened cilia were also detected, reminiscent of abnormalities observed in certain cases of primary ciliopathies^17,49^. We documented by immunofluorescence a thinning of the ciliary layer as early as 2 dpi, associated to a layer of viral spike protein at the base of motile cilia. SARS-CoV-2 particles were not released from cilia, but rather from deciliated areas, as indicated by the minimal colocalization between the spike and β-tubulin markers. Clusters of viral particles could be detected on microvilli, consistent with findings suggesting that SARS-CoV-2 could induce the formation of actin-based filaments in transformed cells^50^. The absence of viral particles within cilia may result from the restricted protein access imposed by the transition zone at the base of motile cilia^51^. Access of cellular proteins into the ciliary axoneme is limited to those bound by the intraflagellar transport machinery, which likely prevents the import of viral proteins. Therefore, the destruction of motile cilia by SARS-CoV-2 seems mediated by an indirect mechanism, rather than by direct viral production within these structures.

TEM imaging confirmed ciliary axoneme loss but also revealed the presence of misoriented basal bodies that had lost plasma membrane docking in productively infected cells. Rootlets, which normally anchor basal bodies into the cytoplasm, were sometimes found isolated, suggesting that basal bodies may themselves detach and/or depolymerize. The presence of basal bodies around or inside vacuoles suggested that these organelles underwent intravacuolar degradation. The Foxj1 transcription factor is known to be required for the docking of basal bodies to the plasma membrane during multiciliogenesis^52^. Patients with inborn heterozygous mutations in the *FOXJ1* gene show a motile ciliopathy with hydrocephalus and a chronic destructive airway disease^53^. Strikingly, airway epithelial cells from these patients show a marked reduction in number of motile cilia and a mislocalization of basal bodies in the cytoplasm and around vacuoles, similar to our findings in SARS-CoV-2-infected epithelia. That Foxj1 protein levels were downregulated upon SARS-CoV-2 infection could therefore account for the mislocalization of basal bodies and the loss of axoneme elongation. The Foxj1 protein half-life was recently shown to be dynamically regulated through ubiquitination and proteasomal degradation^33^. A post-transcriptional mode of regulation helps explain the observation of an early loss of the Foxj1 protein, prior to a decrease in *FOXJ1* transcripts. The late decrease in transcripts encoding ciliogenesis regulators (*FOXJ1*, *RFX3*) and ciliary components (*DNAH7*) may in part result from the death of ciliated cells detectable at a more advanced stage of infection. Taken together our findings are consistent with an initial phase of multiciliated cell dedifferentiation triggered by the early downregulation of Foxj1, compounded by multiciliated cell death at a more advanced stage of infection. Of note, RSV and mouse parainfluenza virus have also been shown to induce a downregulation of Foxj1 expression in infected ciliated cells^37^, suggesting a preferential targeting of this transcription factor by viral mechanisms that subvert mucociliary clearance. More broadly, our findings are compatible with RNAseq studies of SARS-CoV-2-infected epithelial cell cultures^22,54^ and of mucosal samples from COVID-19 patients^55^ that reported a downregulation of transcripts involved in cilia structure and function.

SARS-CoV-2 infection also induced a significant IFN response, that was primarily driven by type III IFN. IFN-λ was abundantly secreted at 4 dpi in the apical and basal compartments of infected reconstructed epithelia. In contrast, IFN-β secretion was minimal, and IFN-α secretion remained undetectable even after using a high-sensitivity digital ELISA for detection. These observations fit with the notion that type III IFN is the dominant antiviral cytokine in epithelia, enabling a localized response mediated directly by epithelial cells, prior to the infiltration of immune cells^56^. It was noteworthy, however, that IFN-λ was secreted mostly from day 4 post-infection onward, while viral RNA production had already shown a 2-log increase by day 2. This delay in the induction of the antiviral response suggests a “too little too late” scenario, where antiviral mechanisms are overwhelmed and subverted by active viral replication. Our findings are compatible with studies showing a limited and/or delayed IFN induction by SARS-CoV-2 as compared to other respiratory viruses, in primary epithelial cells from the airways and the intestine^10,57–59^. These results do not rule out the potential for IFN treatment, as IFN may still exert an antiviral effect if administered early. We observed that pretreatment with IFN-β or IFN-λ markedly decreased SARS-CoV-2 replication in our bronchial epithelium model. These findings are in agreement with studies in various culture systems documenting the inhibitory effect of both type I and type III IFN on SARS-CoV-2 replication, when treatment occurs before the stage of massive viral replication^57,58,60–62^. In contrast, studies in animal models suggest that, at an advanced stage of infection, IFN may contribute to the decline of respiratory function, possibly by worsening inflammation^63^ and impairing epithelial cell regeneration^64,65^. Therefore, the window of opportunity for IFN treatment may have to be carefully defined in randomized controlled trials.

In conclusion, we demonstrate that SARS-CoV-2 induces multiciliated cell dedifferentiation, leading to a rapid loss of the ciliary layer, and resulting in impaired mucociliary clearance. Intrinsic epithelial defense mechanisms likely occur too late to prevent cilia loss. These findings highlight a pathogenic mechanism that may promote viral spread in the respiratory tree and increase the risk of secondary infections in COVID-19 patients.

## Methods

### SARS-CoV-2 infection of reconstructed human bronchial epithelia

MucilAir^TM^, corresponding to reconstructed human bronchial epithelium cultures differentiated in vitro for at least 4 weeks, were purchased from Epithelix (Saint-Julien-en-Genevois, France). Cultures were maintained in air/liquid interface (ALI) conditions in transwells with 700 µL of MucilAir^TM^ medium (Epithelix) in the basal compartment, and kept at 37 °C under a 5% CO_2_ atmosphere.

For SARS-CoV-2 infection, the apical side of ALI cultures was washed 20 min at 37 °C in Mucilair^TM^ medium to remove mucus. Cells were then incubated with 10^6^ plaque-forming units (pfu) of the isolate BetaCoV/France/IDF00372/2020 (EVAg collection, Ref-SKU: 014V-03890; kindly provided by S. Van der Werf). The viral input was diluted in DMEM medium to a final volume 150 µL, and left on the apical side for 4 h at 37 °C. Control wells were mock-treated with DMEM medium (Gibco) for the same duration. Viral inputs were removed by washing twice with 200 µL of PBS (5 min at 37 °C) and once with 200 µL Mucilair^TM^ medium (20 min at 37 °C). The basal medium was replaced every 2–3 days. Apical supernatants were harvested every 2–3 days by adding 200 µL of Mucilair^TM^ medium on the apical side, with an incubation of 20 min at 37 °C prior to collection.

For viral inhibition by interferons, cultures were treated with IFN-β at 1000 U/mL or with a mixture of IFN-λ1 and IFN-λ3 at 1 µg/mL each. Interferons were added to the basal compartment 1 day prior to infection, and then added every 2–3 days to both the apical and basal compartments throughout the infection.

### SARS-CoV-2 infection of Syrian hamsters

Male Syrian hamsters (Mesocricetus auratus) of 5–6 weeks of age (average weight 60–80 g) were purchased from Janvier Laboratories and handled under specific pathogen-free conditions, according to the French legislation and to the regulations of Pasteur Institute Animal Care Committees, in compliance with the European Communities Council Directives (2010/63/UE, French Law 2013–118, February 6, 2013). The Animal Experimentation Ethics Committee (CETEA 89) of the Pasteur Institute approved this study (200023; APAFIS#25326-2020050617114340 v2) before experiments were initiated. Hamsters were housed by groups of four animals, with ad libitum access to water and food. Animals were manipulated in class III safety cabinets in the Pasteur Institute animal facilities accredited by the French Ministry of Agriculture for performing experiments on live rodents. Before any manipulation, animals underwent an acclimation period of one week. All animals were handled in strict accordance with good animal practice.

Animal infections were performed as described with few modifications 34. Briefly, the animals were anesthetized with an intraperitoneal injection of ketamine (200 mg/kg) and xylazine (10 mg/kg). Hundred microliters of physiological solution containing 6 × 10^4^ pfu of SARS-CoV-2 (BetaCoV/France/IDF00372/2020) were administered intranasally. Mock-infected animals received the physiological solution only. The animals were followed-up on a daily basis and euthanized at day 4 post-infection, when the tracheas were collected, immediately frozen at −80 °C or formalin-fixed after transcardial perfusion with a physiological solution containing heparin (5 × 10^3^ U/ml, Sanofi), followed by 4% paraformaldehyde (PFA).

### Viral RNA quantification

#### Reconstructed bronchial epithelia

viral RNAs were extracted from 20 µL of apical and basal culture supernatants using the Quick-RNA Viral 96 kit (Zymo) following manufacturer instructions. SARS-CoV-2 RNA was quantified in a final volume of 5 μL per reaction in 384-well plates using the Luna Universal Probe One-Step RT-qPCR Kit (New England Biolabs) with SARS-CoV-2 N-specific primers (Forward 5’-TAA TCA GAC AAG GAA CTG ATT A-3’; Reverse 5’-CGA AGG TGT GAC TTC CAT G-3’) on a QuantStudio 6 Flex thermocycler (Applied Biosystems). Standard curve was performed in parallel using purified SARS-CoV-2 viral RNA.

#### Hamster tissues

Total RNA was extracted from frozen tracheas using the Direct-zol RNA MicroPrep Kit (R2062, Zymo Research) and reverse transcribed to first strand cDNA using the SuperScript™ IV VILO™ Master Mix (Invitrogen). qPCR was performed in a final volume of 20 μL per reaction in 96-well PCR plates using a thermocycler (7500t real-time PCR system, Applied Biosystems). Briefly, 5 μL of cDNA (25 ng) was added to 15 μL of a master mix containing 10 μL of Power SYBR green mix (4367659, Applied Biosystems) and 5 μL of nuclease-free water with the nCoV_IP2 primers (Forward 5’-ATG AGC TTA GTC CTG TTG-3’; Reverse 5’-CTC CCT TTG TTG TGT TGT-3’).

### Cellular RNA quantification

Cellular RNA expression was measured by real-time quantitative PCR. The epithelial cultures were washed in cold PBS and then lyzed in 150 µL of Trizol reagent (Thermofisher scientific) added to the apical side of the insert. RNAs were purified using the Direct-zol miniprep kit (ZR2080, Zymo Research). Transcripts of genes of interest (*FOXJ1, DNAH7, RFX3, FOXA2, TUBB4A, KRT5, MUC5AC*) were amplified in a final volume of 5 μL per reaction in 384-well plates using the Luna Universal Probe One-Step RT-qPCR Kit (New England Biolabs) with Quantitect primers (Qiagen) on a QuantStudio 6 Flex thermocycler. RT-qPCR results were normalized to the mean expression of four reference genes (GAPDH, TFRC, ALAS1, RLP13) to compute relative gene expression. The list of primers used is reported in Supplementary Table 1.

### TCID50 quantification

Culture supernatants were thawed and serial diluted (10-fold) from 10^−1^ to 10^−8^ in DMEM. Six replicates of viral dilutions (50 µL each) were seeded in flat bottom 96-well plates and mixed with 12,000 VeroE6 cells in DMEM-3% FBS (150 µL). After 5 days at 37 °C, cells were fixed in 4% PFA for 30 min at room temperature (RT). PFA was then replaced by a crystal violet solution for 15 min at RT and rinsed with water. The TCID50 was determined as the lowest viral concentration inducing cell lysis (no crystal violet staining) in 50% of the six replicates.

### Trans-epithelial electrical resistance (TEER) measurement

The apical side of transwell cultures was washed for 20 min at 37 °C in Mucilair^TM^ medium. Transwell were then transferred in a new 24-well plate and DMEM medium was added to both the apical (200 µL) and basal (700 µL) sides. The TEER was then measured using an Evom3 ohmmeter (World Precision Instruments).

### Epithelium permeability assay

The apical side of transwell cultures was washed for 20 min at 37 °C in Mucilair^TM^ medium, and transwell were then transferred in a new 24-well plate. Samples were then quickly rinsed with PBS on both apical and basal sides, and then and incubated in DMEM without phenol-red (Gibco). Dextran-FITC (4 kDa, Sigma–Aldrich) was prepared at 1 mg/mL in DMEM without phenol-red, and 200 µL were added on the apical side. After 30 min at 37 °C, the basal medium was harvested, and FITC fluorescence was measured using an EnSpire luminometer (Perkin Elmer). Medium alone and a Dextran-FITC solution (10 ng/mL) were used as negative and positive controls, respectively.

### LDH cytotoxicity assay

Diluted culture supernatants (1:25) were pretreated with Triton-X100 1% for 2 h at RT for viral inactivation. Lactate dehydrogenase (LDH) dosage was performed using the LDH-Glo™ Cytotoxicity Assay kit (Promega) following manufacturer’s instructions. Luminescence was measured using an EnSpire luminometer (Perkin Elmer).

### Cytokine measurements

Culture supernatants were pretreated with Triton-X100 1% for 2 h at RT for viral inactivation. IFN-β concentrations were quantified with a Simoa digital ELISA developed with Quanterix Homebrew kits as previously described^66^. Briefly, the 710322-9 IgG1-κ, mouse monoclonal antibody (PBL Assay Science) was used as a capture antibody to coat paramagnetic beads (0.3 mg/mL), the 710323-9 IgG1-κ mouse monoclonal antibody (PBL Assay Science) was biotinylated (biotin/antibody ratio = 40/1) and used as the detector antibody, and recombinant IFN-β protein (PBL Assay Science) was used as a reference to quantify IFN-β concentrations. The limit of detection (LOD) for IFN-β was 1.7 pg/mL. IFN-α2 was detected with a Simoa assay with a LOD of 2 fg/mL, as previously described^11^. Additional cytokines were measured with a commercial Legendplex bead-based immunoassay (Biolegend), following manufacturer’s instructions. Samples were analyzed on a LSRII flow cytometer (BD Biosciences).

### Immunofluorescence staining

#### Reconstructed epithelia

MucilAir^TM^ cultures were washed twice with PBS, fixed in 4% PFA for 30 min at RT, washed again twice with PBS and stored in PBS at 4 °C until staining. Transwell membranes pieces were cut using a scalpel blade and staining steps were performed at RT on membrane pieces placed in 10 µL drops on parafilm.

For surface SARS-CoV-2 spike staining, membranes were blocked in PBS—0.1% Tween—1% BSA—10% FBS—0.3 M glycine for 30 min, then incubated with mouse-anti-Spike antibody (kindly provided by N. Escriou, Institut Pasteur) at 1 µg/mL in PBS—0.1% Tween—1% BSA for 30 min, followed by three washes of 5 min in PBS. Secondary anti-mouse antibody conjugated to AF488 (A-11001; Invitrogen) or AF555 (A-21422; Invitrogen) was added at 1:400 in PBS—0.1%-Tween—1%BSA for 30 min, followed by three washes of 5 min in PBS and fixation in 4% PFA for 30 min at RT.

For intracellular staining, samples were first permeabilized with PBS—0.5% Triton for 20 min at RT and then blocked in PBS—0.1% Tween—1% BSA—10% FBS—0.3 M glycine for 30 min. Samples were incubated with conjugated primary antibodies diluted in PBS—0.1% Tween—1% BSA for 1 h at RT or overnight at 4 °C, followed by three washes of 5 min in PBS. Samples were counterstained with Hoechst, followed by three washes of 5 min in PBS, and mounted in Fluoromount-G (Invitrogen) before observation with a STELLARIS (Leica Microsystems) or LM710 (Zeiss) confocal microscope.

Primary antibodies used were: rabbit anti-β-IV-tubulin-AF488 (ab204003; Abcam), rabbit anti-β-IV-tubulin-AF647 (ab204034; Abcam), rabbit anti-cytokeratin-5-AF647 (ab193895; Abcam), rabbit anti-mucin 5AC-AF555 (ab218714; Abcam), rabbit anti-ZO-1(40-2200; Invitrogen), and goat anti-Foxj1 (AF3619, Bio Techne). The list of antibodies, with the dilution used for each antibody, is reported in Supplementary Table 2.

#### Hamster tissues

Tracheas were aseptically harvested and fixed one week in 4% PFA, then washed in PBS and dehydrated in 30% sucrose. They were then embedded in O.C.T compound (Tissue-Tek), frozen on dry ice, and cryostat-sectioned into 20-µm-thick sections. Sections were rinsed in PBS, and epitope retrieval was performed by incubating sections for 20 min in citrate buffer (pH 6.0) at 96 °C for 20 min. Sections were blocked in PBS—10% goat serum—4% FBS—0.4% Triton for 2 h at RT, then incubated with conjugated primary antibodies rabbit anti-β-IV-tubulin AF488 (ab204003; Abcam) and rabbit anti-cytokeratin-5-AF647 (ab193895; Abcam) at 1:100 in PBS—4% goat serum—4% FBS—0.4% Triton overnight at 4 °C. Sections were then counterstained with Hoechst, rinsed thoroughly in PBS, and mounted in Fluoromount-G (Invitrogen) before observation with a Zeiss LM710 inverted confocal microscope.

Immunostaining was also done on whole pieces of hamster tracheas. Small samples (4 × 4 mm) of tracheal tissue fixed in 4% PFA were microdissected, washed three times in PBS and blocked in PBS supplemented with 20% normal goat serum and 0.03% triton X-10 for 1 h at RT, then incubated overnight at 4 °C with primary antibodies: mouse-anti-Spike (1:100, provided by N. Escriou, Institut Pasteur) and rabbit conjugated-anti-β-IV-tubulin-AF647 (ab204034; Abcam) in PBS supplemented with 2% BSA. The samples were rinsed three times in PBS, and incubated for 1 h at RT with secondary antibody ATTO-488-conjugated goat anti-mouse (1:500 dilution, in PBS—2% BSA; Sigma–Aldrich). Sample where then mounted in Fluorsave (Calbiochem) and observed with a Zeiss LSM 710 confocal microscope.

### Confocal laser scanning microscopy

MucilAir^TM^ stained samples were visualized primarily with a STELLARIS 8 inverted microscope (Leica Microsystems, Mannheim, Germany). A super continuum white light laser tunable between 440 and 790 nm was used for excitation and focused through an HC PL APO CS2 ×40 NA 1.1 water immersion or a HC PL APO CS2 ×63 NA 1,3 glycerol immersion objective. Emission signals were captured with Power HyD Detectors. The system was controlled with Leica Application Suite (LAS) X v4.1 software. 3D images were directly processed using LAS X 3D module. The excitation wavelength and detection window are given in parentheses for each fluorophore: Hoeschst (405 nm; 410–470 nm); AlexaFluor488 (499 nm; 504–533 nm); AlexaFluor555 (553 nm; 558–600 nm); AlexaFluor647 (653 nm; 658–700 nm).

Samples of hamster trachea were imaged with an inverted Zeiss LSM 710 confocal microscope controlled by the ZEN pro 2.3 software. Z-stack images of tissue sections and whole-mount samples were acquired with a Plan Apochromat ×20/0.8 Ph2 M27 lens or a Plan Apochromat ×63/1.4 N.A. oil immersion lens (Carl Zeiss).

### Scanning electron microscopy

Samples were fixed with 2.5% glutaraldehyde in 0.1 M cacodylate buffer for 1 h at RT and then for 12 h (MucilAir^TM^ samples) or one week (hamster trachea samples) at 4 °C to inactivate SARS-CoV-2. Samples were then washed in 0.1 M cacodylate buffer and several times in water, and processed by alternating incubations in 1% osmium tetroxide and 0.1 M thiocarbohydrazide (OTOTO), as previously described^67^. After dehydration by incubation in increasing concentrations of ethanol, samples were critical point dried, mounted on a stub, and analyzed by field emission scanning electron microscopy with a Jeol JSM6700F microscope operating at 3 kV.

### Transmission electron microscopy

Samples were fixed with 2.5% glutaraldehyde in DMEM medium with 10% fetal calf serum for 1 h at RT, and then overnight at 4 °C. Fixed samples were washed three times for 5 min in PBS buffer, post-fixed for 1 h in 1% osmium tetroxyde solution, and rinsed with distilled water. Samples were dehydrated through a graded series of ethanol solutions (25, 50, 75, 95, and 100%) and embedded in epoxy resin. Samples were sectioned with an UC7 Leica ultramicrotome (Leica, Wetzlar, Germany) at a thickness of 70 nm, and transferred on 200 Mesh Square Copper grids coated with Formvar and carbon (FCF-200-Cu50, Delta Microscopy). Samples were then stained with 4% uranyl acetate and counterstained with lead citrate. Images were recorded with a TECNAI SPIRIT 120 kV microscope with a bottom-mounted EAGLE 4 K × 4 K Camera, (ThermoFisher Scientific, Waltham, MA, USA).

### Image analysis

#### Quantitative image analysis of the ZO-1-associated tight junction pattern

The surface of the epithelium contained in each microscopy z-stack was extracted using the Zellige program (C. Trébeau, R. Etournay, manuscript in preparation): the tissue 3D-manifold is determined based on both the detection of intensity maxima along the z direction and the spatial correlation of adjacent maxima along the *x, y* directions, with a maximum *z* difference ∆*z* = ±1 between adjacent maxima. The 3D-manifold is then projected onto a plane. The TissueMiner software was then used to segment and quantify the cell apical surface area and the cell neighbor number^68^. The Wilcoxon test was used to compare the median cell area between conditions. The Welch corrected *t*-test was used to compare the average neighbor number between conditions.

#### Quantifications of β-tubulin+ area and spike+ cells in epithelial cultures

Confocal images of samples stained with anti-β-IV-tubulin and anti-spike antibodies were first imported and visualized by Z-stack projection in the ImarisViewer 9.5.1 software. Images were then thresholded on the tubulin signal (0.06) using the FIJI software v2.1.0, and the tubulin+ area was measured with the area measurement tool. The area was normalized to the total area of the picture. To quantify the percentage of spike+ cells, the numbers of Hoechst-stained nuclei and of spike+ cells were manually counted on maximal intensity projections, and the percentage was computed as (spike+ cell number/nuclei number) × 100.

#### Quantification of β-tubulin depth intensity profile in cilia

Samples were imaged at ×63 magnification using the STELLARIS 8 microscope (Leica Microsystems), and image analysis was carried out using customized scripts written in Python 3.7.

##### Cell segmentation

An ImageJ user-guided tool was developed to extract single cells in the epithelium. Three cell categories were defined: cell from a mock-infected sample (mock), cell expressing the spike antigen in an infected sample (spike+), and bystander cell not expressing the spike antigen in an infected sample (spike−). A dataset of 79 mock, 85 spike+, and 71 spike− cells was extracted from 2 mock samples and 4 SARS-CoV-2-infected samples.

##### Averaged cell intensity profiles

For each cropped cell, the intensities of β-tubulin and spike were averaged along the depth axis. The resulting intensity profiles for all cells were then realigned using as a reference the proximal intensity peak of β-tubulin located just below the plasma membrane. The average intensity profiles of β-tubulin and spike along the cell depth axis was then computed for each of the 3 cell categories.

##### β-tubulin distal to proximal peak intensity ratio

The averaged β-tubulin profiles showed two peaks, with a distal peak corresponding to cilia and a proximal peak located just below the plasma membrane, which was used for realignment. The proximal peak corresponded to the area where basal bodies anchor cilia into the cytoplasm. The ratio between the intensities of the distal peak and the proximal peak was computed for each cell.

##### Colocalization analysis

Images were acquired at the Z level where the spike signal was maximal, corresponding to the area just above the plasma membrane. Images were deconvoluted with the Huygens Professional version 19.04 software (Scientific Volume Imaging, The Netherlands, http://svi.nl). Thresholded Mander’s colocalization coefficients between the β-tubulin and spike markers were then computed using Otsu algorithm^69^.

#### Quantitation of Foxj1 expression in nuclei

Foxj1 expression was quantified in 12 images obtained from *n* = 4 infected epithelial samples at 2 dpi. The apical fluorescence intensity of the Spike marker was used to manually define Spike+ and Spike− regions. To segment Dapi-labeled nuclei, we used the StarDist software^70^, using the “2D_versatile_fluo” model provided by the authors. The Dapi signal was segmented in each slice of the volume, and the 3D nuclei were then reconstructed by merging 2D segmentation labels along the depth axis, using a distance criterion between centroids and overlap criteria between label regions. For each segmented nucleus, the average Foxj1 fluorescence intensity was computed on data normalized using the (1, 99.8)-percentiles for each sample. Each nucleus was then tagged as Foxj1+ if its average Foxj1 intensity was ≥0.2. The ratio of Foxj1+ nuclei to total nuclei was then computed for the Spike+ and Spike− regions of each sample. A minimum of 700 segmented nuclei was analyzed per image.

#### Quantification of basal cell height

##### Tissue level correction

The reconstructed epithelia were grown on inserts that were not perfectly flat, due to experimental constraints. It was necessary to correct for local insert deformation before measuring the height of basal cells within the epithelia. Due to the insert strong autofluorescence in both the spike and cytokeratin-5 channels, insert elevation could be measured by finding the depth value of the maximal intensity of both signals multiplied, defined as *Z*_insert_(*x,y*) = argmax_z_(I_spike_(*x,y*) × I_btub_(*x,y*)). This value provided the insert elevation map used to correct for tissue deformation. The map was blurred with a gaussian filter of sigma=3 to smooth the results and remove possible small noise.

##### Cytokeratin-5 density profile

The cytokeratin-5 pixel density profile was computed by first smoothing the signal using a sigma = 1.5 gaussian blur and then thresholding the signal using Otsu algorithm^69^. The Cytokeratin-5 density profile for each sample was computed along the Z-axis by measuring the ratio of thresholded pixels over total pixels per plan. The profiles were realigned using the 2/3 of maximum intensity and averaged together. The significance of the difference between the profiles obtained from mock and infected samples was evaluated with a Welch *t*-test comparing profile area under the curve, using the trapezoidal numerical integration algorithm.

##### Quantification of ciliated area in SEM images

Ciliated areas in SEM images were selected and masked in the Adobe Photoshop v21.1.3. software, using the object selection tool, which uses machine learning to automatically shrink wrap an object. Selection of each ciliated area was refined manually with the lasso tool. The masked surface was then measured in the Fiji software^71^.

### Mucociliary clearance assay

Experiments were performed at 7 dpi to allow mucus layer reconstitution after infection. 10 µL of 30 µm polystyrene beads (Sigma–Aldrich) diluted 1:3 in PBS were added to the apical side of transwell cultures. Bead movements were recorded at 37 °C in the brightfield channel of a Biostation IMq inverted microscope (Nikon), taking 1 picture every 2 s during 1 min using the Biostation IM v2.21 software. A minimum of 7 fields per sample were recorded within the first 15 min after addition of beads.

For image analysis, beads were first manually dot-marked using pencil tool from the FIJI software. Tracks were generated and analyzed using the TrackMate FIJI plugin v5.2.0^72^. Dot-marked beads were automatically detected with LoG detector (estimated diameter = 20 µm; threshold = 5–8). Tracks were then generated using the Simple LAP tracker (gap-closing max frame gap = 0), adjusting linking max distance (50–200 µm) and gap-closing max distance (15–100 µm) for each field depending on speed and bead density. The resulting tracks statistics included speed, XY movements, and track straightness parameters. Tracks composed of 3 dots of less were removed from the analysis. Examples of track images for Fig. 5 were generated with the Imaris software (Oxford Instruments).

### Statistics and reproducibility

Statistical analyses were performed with the Prism software v8.4.3 (GraphPad) for all figures except for Fig. 6B, C and Supplementary Figs. 1 and 4. The nonparametric Mann–Whitney test was used, except when data were estimated to be normally distributed (Fig. 4E, F, Fig. 5E, F). For Fig. 6B, C and Supplementary Fig. 4, statistical analyses were done in Python 3.7, using the Matplotlib 3.4.2 api for the plots and scipy 1.6.3 for the statistical tests. For Supplementary Fig. 1, statistical analyses were carried out in the TissueMiner software^68^. All Mann–Whitney tests and *t*-tests were two-sided. Statistical significance was assigned when *p* values were <0.05. Box-whisker plots show median (horizontal line), interquartile range (box), and 1.5× the interquartile range (whiskers). The nature of statistical tests and the number of experiments or animals (n) are reported in the figure legends.

Micrographs are representative of *n* = 2 independent experiments for Figs. 1F, 4A, B, 7C, 8B, and Supplementary Figs. 1 A, 1E, 4A, B, 5A–E; of *n* = 3 independent experiments in Figs. 2C, 2F,G, 5A,B, 6A, 8A, and Supplementary Figs. 2C, 3B–D, 6A–C; of *n* = 4 independent experiments in Figs. 2A, B, 3A–E, 4D, and Supplementary Figs. 2B and 3A. The number of replicate images per independent experiment was ≥3.

### Reporting summary

Further information on research design is available in the Nature Research Reporting Summary linked to this article.

## Supplementary information

Supplementary Information

Description of Additional Supplementary Files

Supplementary Movie 1

Supplementary Movie 2

Reporting Summary

## Data Availability

Data supporting the findings of the present study are provided in the article and Supplementary Information files or from the corresponding authors upon reasonable request. Source data are provided with this paper.

## Source data

Source Data

## Code availability

The image analysis scripts used for computing β-tubulin IV, cytokeratin-5, and Foxj1 distributions are available from S.R. upon reasonable request. The Zellige script used to extract the surface of the epithelium in ZO-1 labeled samples is available from R.E. upon reasonable request. The TissueMiner toolkit used to analyze perturbations in epithelial packing is available at: GitHub https://github.com/mpicbg-scicomp/tissue_miner#about and is referenced in ref. ^68^ at 10.7554/eLife.14334.
