# Supplementary material for: SARS-CoV-2 infection induces the dedifferentiation of multiciliated cells and impairs mucociliary clearance
Source: Nat Commun. 2021 Jul 16;12:4354. doi: 10.1038/s41467-021-24521-x (PMC8285531; doi:10.1038/s41467-021-24521-x)
Supplement: Supplementary file 1 — Supplementary Information [file 41467_2021_24521_MOESM1_ESM.pdf]

SARS-CoV-2 infection induces the dedifferentiation of multiciliated cells and impairs mucociliary clearance

Rémy Robinot*, Mathieu Hubert*, Guilherme Dias de Melo, Françoise Lazarini, Timothée Bruel, Nikaïa Smith[4] Sylvain Levallois, Florence Larrous, Julien Fernandes, Stacy Gellenoncourt, Stéphane Rigaud, Olivier Gorgette, Catherine Thouvenot, Céline Trébeau, Adeline Mallet, Guillaume Duménil, Samy Gobaa, Raphaël Etournay, Pierre-Marie Lledo, Marc Lecuit, Hervé Bourhy, Darragh Duffy, Vincent Michel**, Olivier Schwartz**, and Lisa A. Chakrabarti**.

* These authors contributed equally: Rémy Robinot, Mathieu Hubert
** correspondence: vincent.michel@pasteur.fr, olivier.schwartz@pasteur.fr, and chakra@pasteur.fr

SUPPLEMENTARY INFORMATION CONTENT

Supplementary Figure 1: Quantitative image analysis of the ZO-1-associated tight junction pattern
Supplementary Figure 2: SARS-CoV-2 induces a cytopathic effect in reconstructed human bronchial epithelia
Supplementary Figure 3: SARS-CoV-2 occasionally infects basal and secretory cells
Supplementary Figure 4: The viral spike antigen does not colocalize with cilia
Supplementary Figure 5: SARS-CoV-2 infection leads to the accumulation of mislocalized basal bodies
Supplementary Figure 6: Image analysis of basal cells
Supplementary Figure 7: Persistence of a high viral load in long-term infection of a reconstituted bronchial epithelium
Supplementary Table 1: List of primers used for analysis of gene expression by qRT-PCR
Supplementary Table 2: List of antibodies used in the study
Supplementary Movie 1: Mucociliary clearance assay in a mock infected sample
Supplementary Movie 2: Mucociliary clearance assay in SARS-CoV-2 infected sample

____

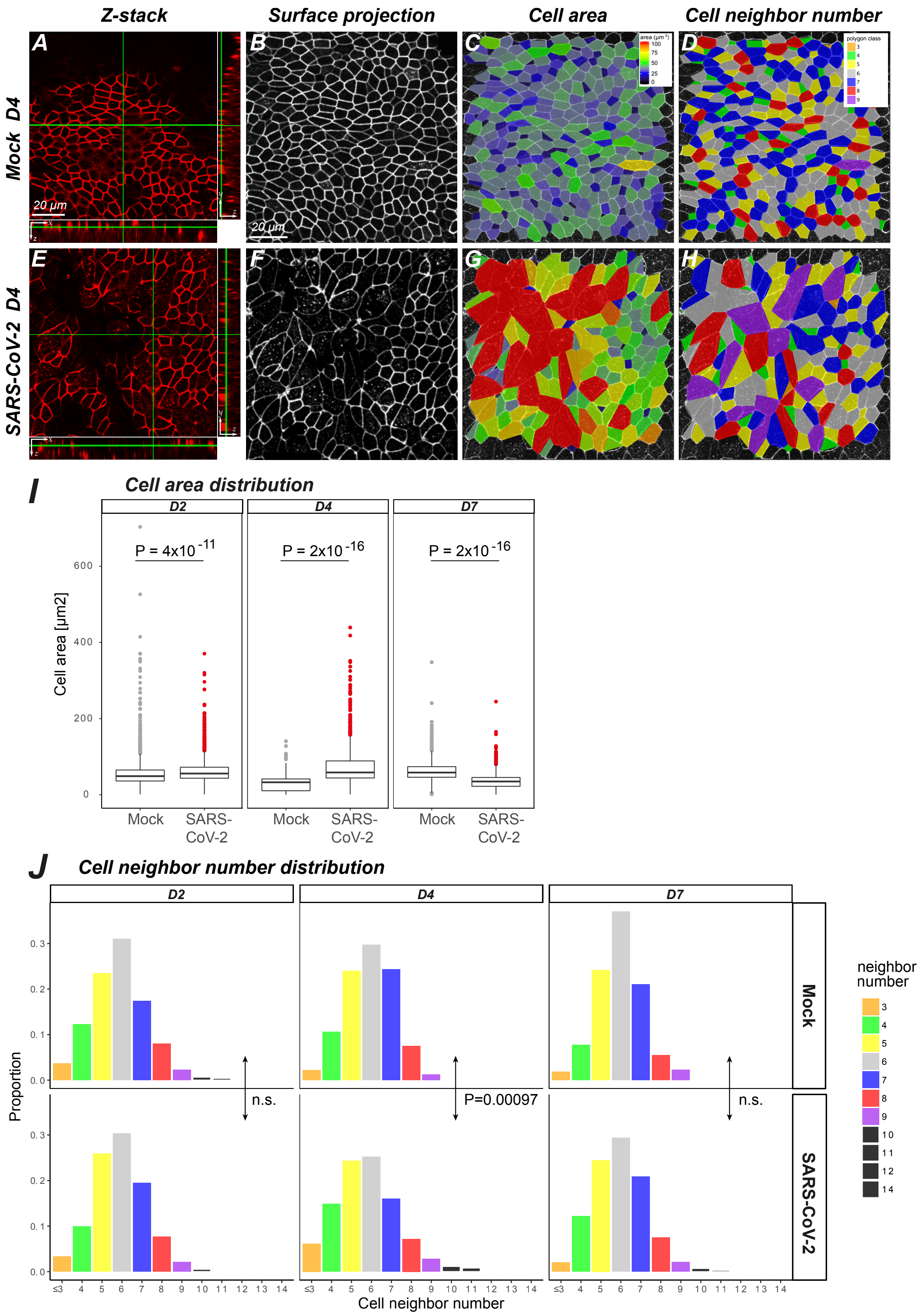

Supplementary Fig. 1: Quantitative image analysis of the ZO-1-associated tight junction pattern

Reconstructed epithelia were labeled with a ZO-1 antibody (red) to detect the tight junction pattern. Confocal images were analyzed with a dedicated software pipeline to quantify cell shape and cell neighbor numbers. **(A-H)** Examples of ZO-1 image analyzes for mock (A-D) and SARS-CoV-2 infected (E-H) epithelia at 4 dpi (n=1 representative experiment out of 2). Original microscopy z-stack labelled with ZO-1 (red) are visualized in (A, E). The surface of the epithelium is extracted and projected onto the (x,y) plane using the Zellige program (C. Trébeau, R. Etournay, in preparation) (B,F). Cell segmentation and subsequent quantifications are performed with the TissueMiner software using the data processed with Zellige. The cell area size (C, G) is color-coded in the range of 0-100 µm2. The cell neighbor number pattern (D, H) is color-coded according to the number of neighbors. Scale bars measure 20 µm. **(I)** Comparison of cell area distribution in mock and infected epithelia at 2, 4, and 7 dpi (≥475 cells examined per time point over n=2 independent experiments). The Wilcoxon test with continuity correction was used to compare the median cell area between conditions. Box plots show median and interquartile range (box) and 1.5x the interquartile range (whiskers), with outliers shown. **(J)** Comparison of the distribution of cell neighbor numbers in mock and infected epithelia at 2, 4, and 7 dpi (same n as in I). The Welch corrected t-test was used to compare the average neighbor number between conditions (P values reported next to vertical arrows).

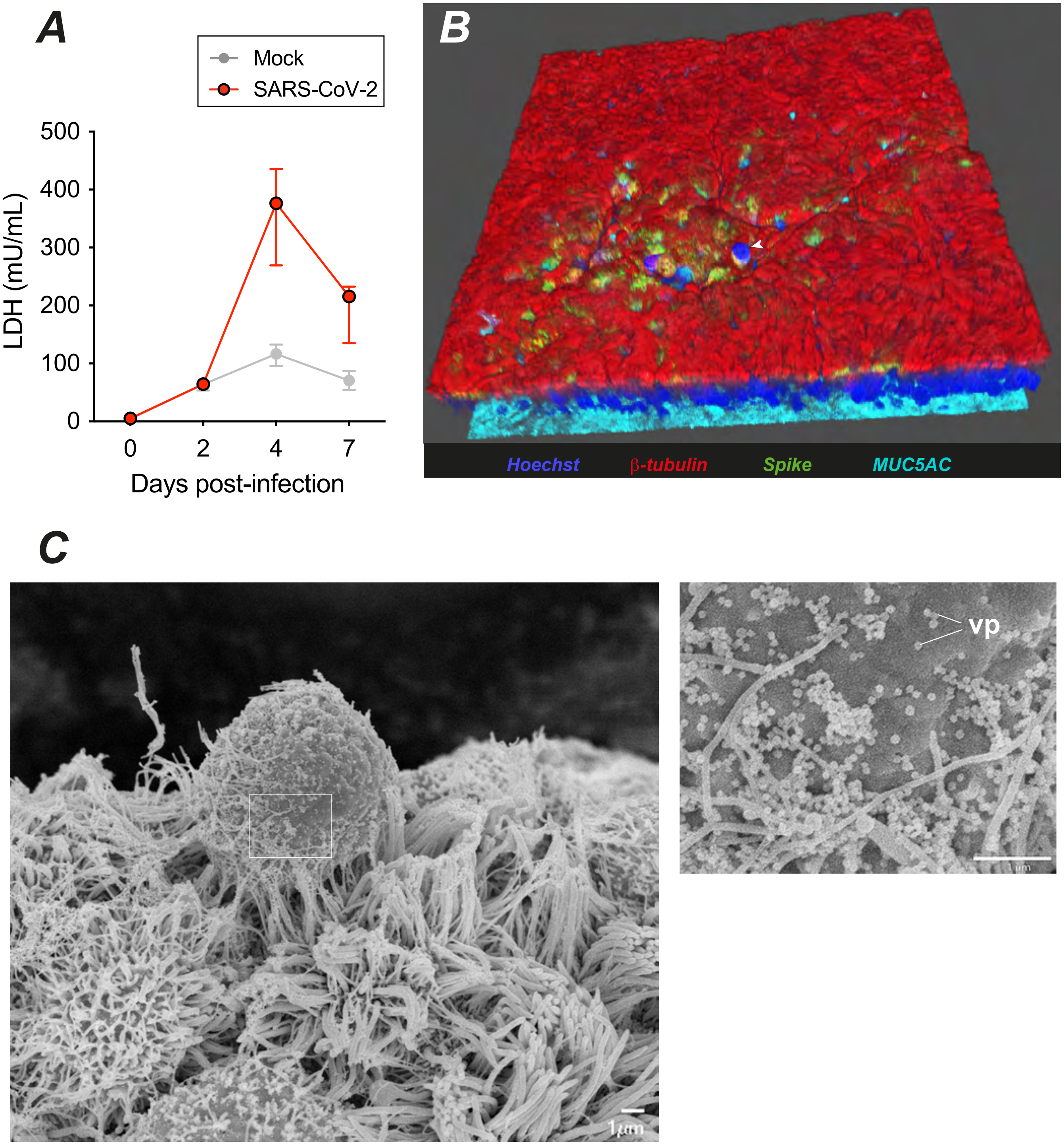

Supplementary Fig. 2: SARS-CoV-2 induces a cytopathic effect in reconstructed human bronchial epithelia
(A) Quantification of the lactate dehydrogenase enzyme (LDH) activity in apical supernatants to evaluate cell death in mock and SARS-CoV-2 infected epithelia (median ± IQR for n=2 independent experiments, with 1 to 8 replicates per experiment). **(B)** 3D confocal image of a SARS-CoV-2 infected epithelium showing nucleus extrusions (Hoechst, blue) above the layer of ciliated cells (β-tubulin+, red) in an area with viral production (spike+, green), while goblet cells (MUC5AC, cyan) do not appear infected. **(C)** Scanning electron microscopy (SEM) image of a rounded extruded cell (left). An enlargement shows the presence of viral particles (vp) at the cell membrane (right).

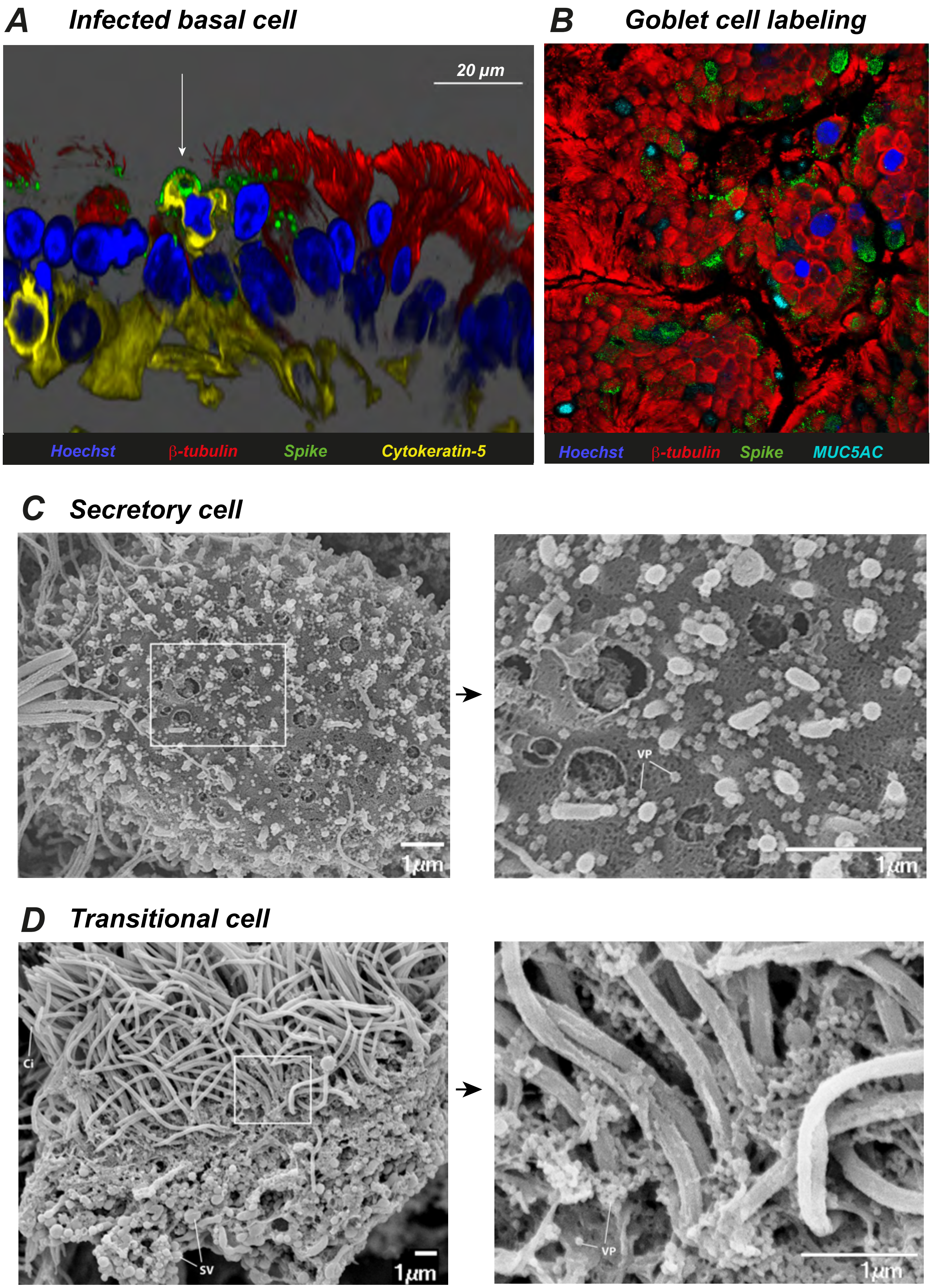

Supplementary Fig. 3: SARS-CoV-2 occasionally infects basal and secretory cells
(A) Confocal image of a rare infected basal cell (cytokeratin 5+ yellow and spike+ green; arrowhead) in a reconstructed epithelium at 2 dpi. **(B)** Goblet cells (MUC5AC+, cyan) in a reconstructed epithelium at 2 dpi do not express the spike antigen (green). (A, B) Ciliated cells are labelled with β-tubulin (red) and nuclei with Hoechst (blue). **(C)** SEM image of a secretory cell with characteristic membrane pores and abundant viral particles (vp) at its surface. An enlargement of the framed area is shown to the right. **(D)** SEM image of a transitional cell showing both secretory vesicles (sv) and cilia (ci). The enlargement to the right shows the presence of viral particles (vp) at the cell surface.

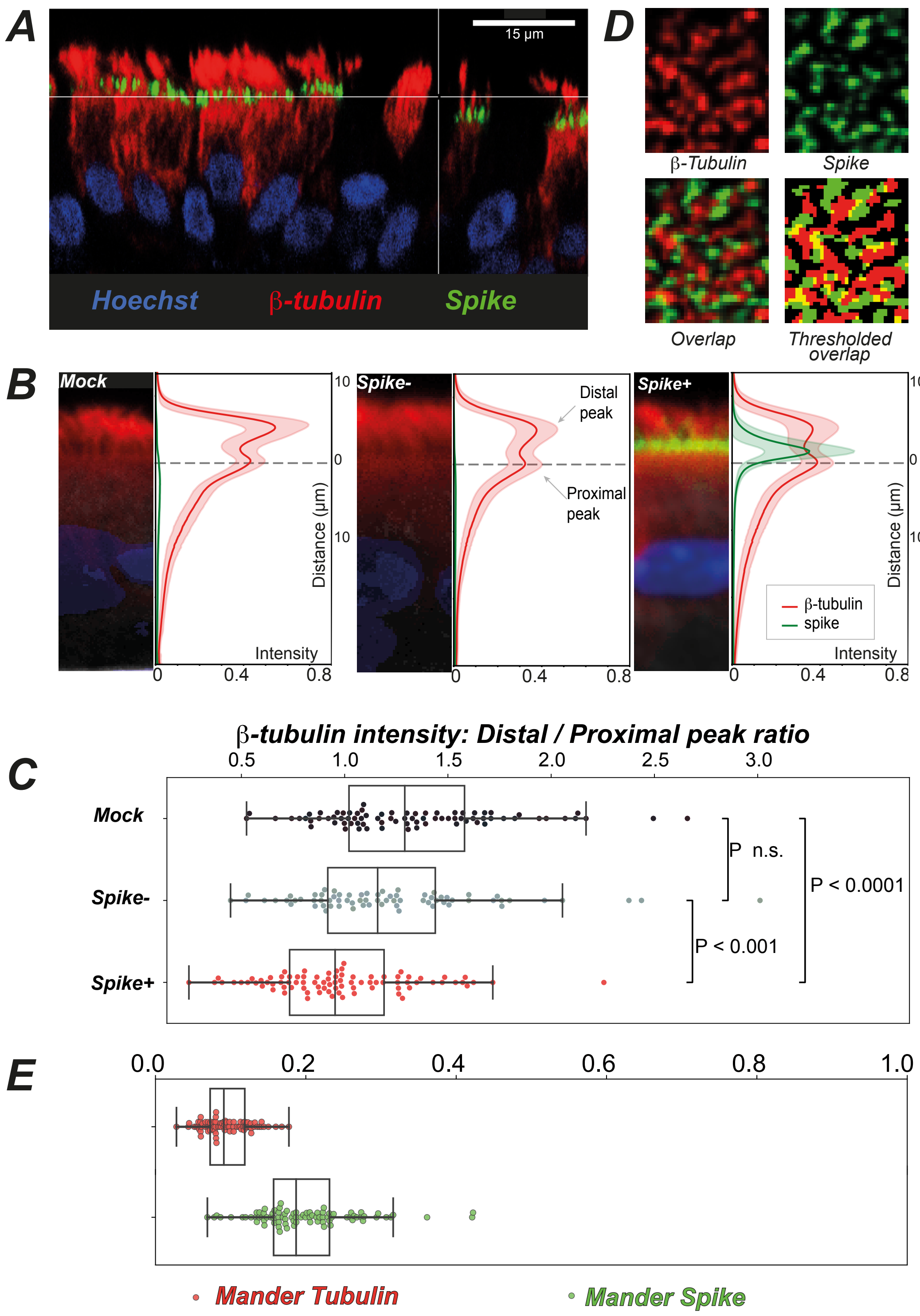

Supplementary Fig. 4: The viral spike antigen does not colocalize with cilia

A) Example of a confocal image showing the distribution of β-tubulin IV (red) and SARS-CoV-2 spike (green) along the depth axis of a reconstructed epithelium at 2 dpi. Nuclei are labeled with Hoechst (blue). **(B)** Distribution of β-tubulin IV (red) and SARS-CoV-2 spike (green) immunofluorescence (IF) labeling at 2 dpi. Representative IF images projected on the Z (depth) axis are shown next to averaged profiles of β-tubulin intensities. Profiles were measured along the Z axis for cells from 79 cells from an uninfected sample (Mock) and from 71 spike- cells and 85 spike+ cells from an infected sample, in n=1 experiment. The mean±SD of β-tubulin intensity is reported. **(C)** Ratios of β-tubulin distal to proximal peak intensities for 3 cell categories were compared with Welch corrected t-tests (same n as in B). Box plots show median and interquartile range (box) and 1.5x the interquartile range (whiskers), with outliers shown.

(D, E) Analysis of spike and β-tubulin colocalization in infected cells at 2 dpi. **(D)** shows a representative infected cell viewed in the x,y plane at membrane level. Little overlap is seen between cilia (red spots) and spike+ areas (green spots), as shown by the limited extent of the yellow area. A thresholded image (bottom right) was used to compute colocalization coefficients. **(E)** Measurement of Mander colocalization coefficient for n=85 spike+ cells for n=1 experiment. The Mander Tubulin coefficient measures the fraction of β-tubulin+ intensity that is located in spike+ pixels. Conversely, the Mander Spike coefficient measures the fraction of spike intensity that is located in β-tubulin+ pixels. Both coefficients are low, indicating minimal colocalization of the two markers. Box plots: same definition as in C.

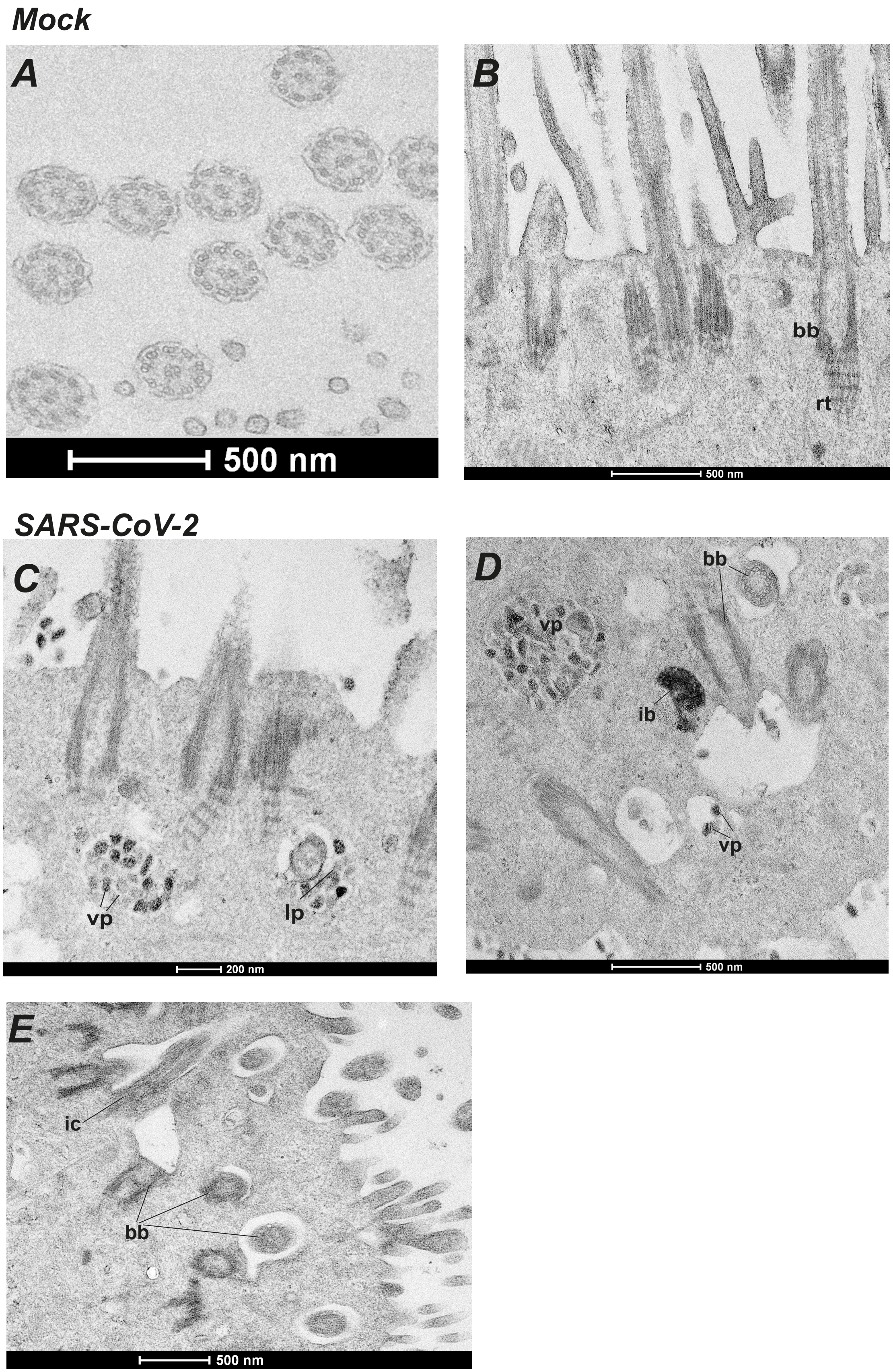

Supplementary Fig. 5: SARS-CoV-2 infection leads to the accumulation of mislocalized basal bodies
(A, B) TEM images of motile cilia in a mock-infected reconstructed bronchial epithelium. (A) Motile cilia seen in transverse section. In each cilium, 9 peripheral microtubule doublets and a central microtubule pair are visible. (B) Motile cilia seen in longitudinal section: elongated axonemes are anchored beneath the plasma membrane through basal bodies (bb) prolonged by rootlets (rt). **(C-E)** TEM images of infected ciliated cells at 4 dpi showing viral particles (vp) and large particles (lp) inside vesicles (C), mislocalized basal bodies (bb) close to or engulfed in vacuoles (D), electron-dense inclusion bodies (ib, D), and a rare internalized cilium (ic, E).

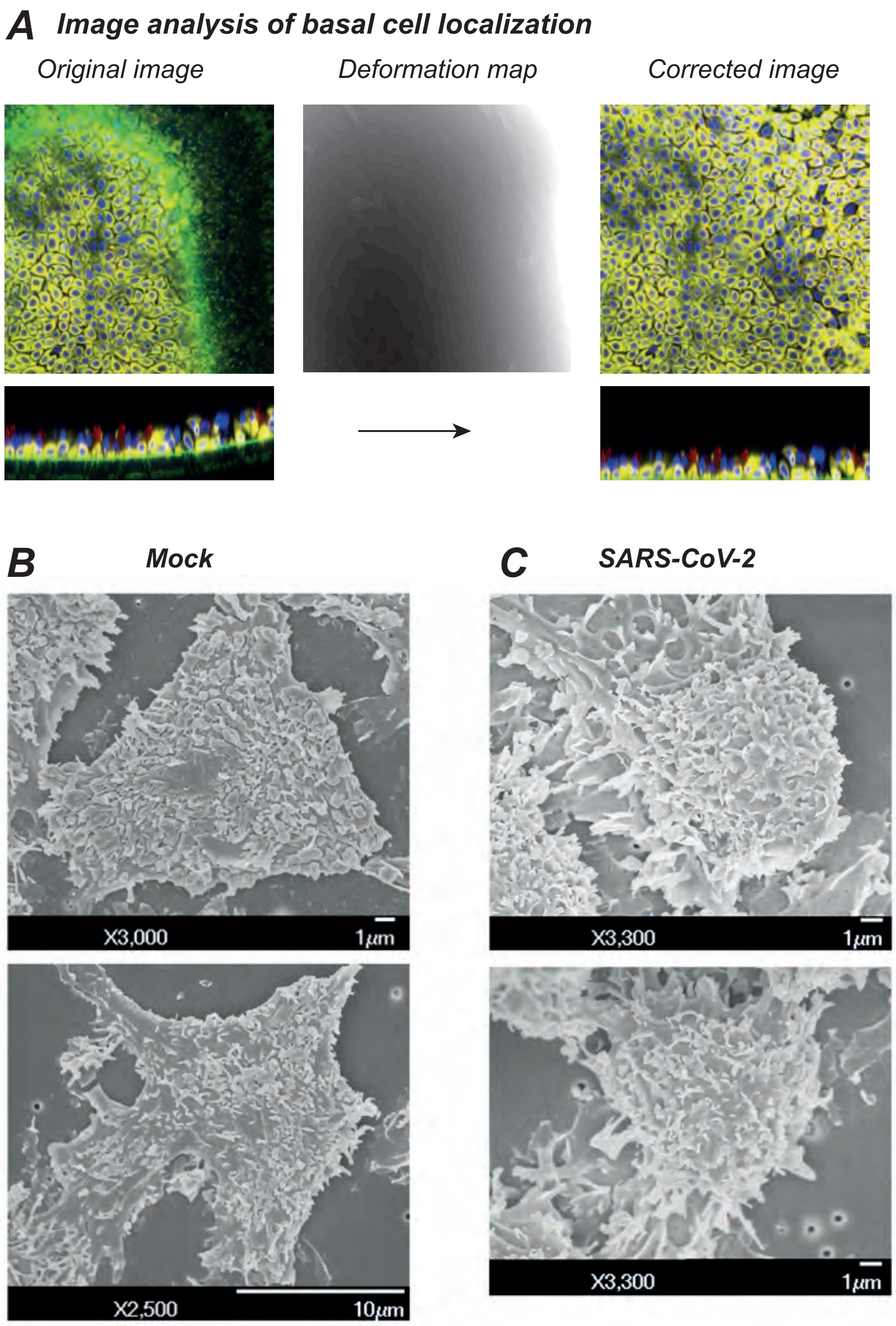

Supplementary Fig. 6: Image analysis of basal cells

(A) Procedure for image correction of local height deformation: the tissue sample was grown on an insert that was not entirely flat due to experimental constraints (left panel, top). The height of basal cells (cytokeratin-5+, yellow) is skewed by insert deformation, as shown in an orthogonal projection (left panel, bottom). The insert is visible on confocal images due to autofluorescence, so that an insert deformation map can be generated (middle panel). The map is then used to correct images for tissue deformation (right panel). Ciliated cells are labeled with β-tubulin (red) and nuclei with Hoechst (blue). **(B, C)** SEM images of basal cells in mock (B) and infected (C) epithelia at 7 dpi. Two representative images are shown for each condition.

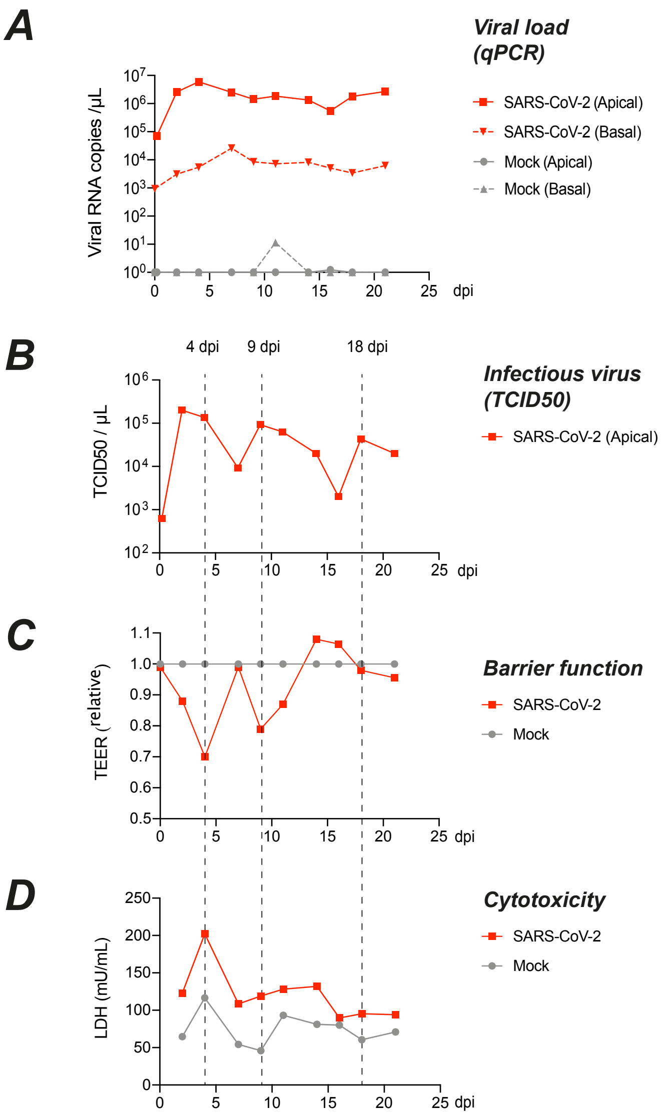

Supplementary Fig. 7: Persistence of a high viral load in long-term infection of a reconstituted bronchial epithelium

(A) SARS-CoV-2 viral RNA copy numbers measured in the apical and basal compartments over a 21 day period (dpi: days post-infection; n=1 experiment). **(B)** Infectious viral titers measured in the apical supernatant over a 21 day period. TCID50 were measured by infecting Vero-E6 cells with serial dilutions of apical culture supernatant. **(C)** Barrier function was evaluated by the trans-epithelial electrical resistance (TEER) measured in the infected sample and normalized to values in the corresponding mock-infected sample. **(D)** Quantification of lactate dehydrogenase enzyme (LDH) activity in apical supernatants to evaluate cell death in SARS-CoV-2 and mock infected epithelia. (B-D) Peaks in infectious virus release are marked with dashed lines. Peaks in infectious virus approximately coincided with minima in relative TEER values and increases in LDH activity.

GENE TARGET	PRIMER SEQUENCE or REFERENCE
ALAS1	QT00073122 (Qiagen)
DNAH7	QT00016338 (Qiagen)
FOXA2	QT00212786 (Qiagen)
FOXJ1	QT01000797 (Qiagen)
GAPDH	QT00273322 (Qiagen)
KRT5	QT00053046 (Qiagen)
MUC5AC	QT01329615 (Qiagen)
RLP13	For : 5’-AGG CAT CAA CAT TTC TGG CA-3’<br>Rev : 5’-CCA TCC GCT TTT TCT TGT CG-3’
RFX3	QT00025970 (Qiagen)
SARS-CoV-2 N	For : 5'-CGA AGG TGT GAC TTC CAT G-3'<br>Rev : 5'-TAA TCA GAC AAG GAA CTG ATT A-3'
TFRC	QT00094850 (Qiagen)
TUBB4A	QT01668632 (Qiagen)

Supplementary Table 1: List of primers used for analysis of gene expression by qRT-PCR
For: forward primer
Rev: reverse primer

ANTIBODY	DILUTION	SOURCE	REFERENCE
Rabbit anti-β-IV-tubulin AF488	1:100	Abcam	ab204003
Rabbit anti-β-IV-tubulin AF647	1:100	Abcam	ab204034
Rabbit anti-cytokeratin 5 AF647	1:100	Abcam	ab193895
Rabbit anti-mucin 5AC AF555	1:50	Abcam	ab218714
Rabbit anti-ZO-1	1:100	Invitrogen	40-2200
Mouse anti-SARS-CoV-2 spike #702	1:100	In house	N/A
Goat anti-FoxJ1	1:100	R&D Systems	AF3619
Secondary anti-mouse AF488	1:400	Invitrogen	A-11001
Secondary anti-mouse AF555	1:400	Invitrogen	A-21422
Secondary anti-goat AF488	1:400	Invitrogen	A-11055

Supplementary Table 2: List of antibodies used in the study.
The dilutions used for immunofluorescence labeling are reported in the second column. The antibody supplier and the supplier reference are reported in the third and fourth columns, respectively. N/A: not available.
