# Supplementary material for: SARS-CoV-2 infection induces the dedifferentiation of multiciliated cells and impairs mucociliary clearance
Source: Nat Commun. 2021 Jul 16;12:4354. doi: 10.1038/s41467-021-24521-x (PMC8285531; doi:10.1038/s41467-021-24521-x)
Supplement: Supplementary file 2 — Description of Additional Supplementary Files [file 41467_2021_24521_MOESM2_ESM.pdf]

Robinot et al.

LEGENDS TO THE SUPPLEMENTARY MOVIES

Supplementary Movie 1: **Mucociliary clearance assay in a mock infected sample**

The movement of 30 µm beads at the surface of an uninfected reconstructed bronchial epithelium is shown in the first half of the movie. The resulting tracks, color-coded for speed, are shown in the second half of the movie.

Supplementary Movie 2: **Mucociliary clearance assay in SARS-CoV-2 infected sample**

The movement of 30 µm beads at the surface of a SARS-CoV-2 infected reconstructed bronchial epithelium at 7 dpi is shown in the first half of the movie. The resulting tracks, color-coded for speed, are shown in the second half of the movie.
