# Supplementary material for: SARS-CoV-2 infection induces the dedifferentiation of multiciliated cells and impairs mucociliary clearance
Source: Nat Commun. 2021 Jul 16;12:4354. doi: 10.1038/s41467-021-24521-x (PMC8285531; doi:10.1038/s41467-021-24521-x)
Supplement: Supplementary file 5 — Reporting Summary [file 41467_2021_24521_MOESM5_ESM.pdf]

nature research

Corresponding author(s): V. Michel, O. Schwartz, L.A. Chakrabarti

Last updated by author(s): 07/06/2021

Reporting Summary

Nature Research wishes to improve the reproducibility of the work that we publish. This form provides structure for consistency and transparency in reporting. For further information on Nature Research policies, see our Editorial Policies and the Editorial Policy Checklist.

Statistics

For all statistical analyses, confirm that the following items are present in the figure legend, table legend, main text, or Methods section.

n/a	Confirmed	
☐	☒	The exact sample size (*n*) for each experimental group/condition, given as a discrete number and unit of measurement
☐	☒	A statement on whether measurements were taken from distinct samples or whether the same sample was measured repeatedly
☐	☒	The statistical test(s) used AND whether they are one- or two-sided<br>*Only common tests should be described solely by name; describe more complex techniques in the Methods section.*
☒	☐	A description of all covariates tested
☐	☒	A description of any assumptions or corrections, such as tests of normality and adjustment for multiple comparisons
☐	☒	A full description of the statistical parameters including central tendency (e.g. means) or other basic estimates (e.g. regression coefficient) AND variation (e.g. standard deviation) or associated estimates of uncertainty (e.g. confidence intervals)
☐	☒	For null hypothesis testing, the test statistic (e.g. *F*, *t*, *r*) with confidence intervals, effect sizes, degrees of freedom and *P* value noted<br>*Give P values as exact values whenever suitable.*
☒	☐	For Bayesian analysis, information on the choice of priors and Markov chain Monte Carlo settings
☒	☐	For hierarchical and complex designs, identification of the appropriate level for tests and full reporting of outcomes
☒	☐	Estimates of effect sizes (e.g. Cohen's *d*, Pearson's *r*), indicating how they were calculated

Our web collection on statistics for biologists contains articles on many of the points above.

Software and code

Policy information about availability of computer code

Data collection	Confocal images made with the STELLARIS 8 microscope (Leica Microsystems) were acquired with the Leica Application Suite (LAS) X v4.1 software and 3D images were directly processed using the LAS X 3D module. Confocal images made with the LSM 710 microscope (Zeiss) were acquired using the ZEN pro 2.3 software. In mucociliary clearance assays, movies were generated on a Biostation IMq inverted microscope (Nikon), using the Biostation IM v2.21 software.
Data analysis	Confocal images were imported in the ImarisViewer 9.5.1 software to generate maximum projections and orthogonal slices, which were then exported as .tif files for inclusion in figures. Custom scripts were developed in Python 3 to analyze the distribution of the beta-tubulin IV, cytokeratin 5, and Foxj1 markers in 3D confocal images. For colocalisation analyses, images were deconvoluted with the Huygens Professional version 19.04 software (Scientific Volume Imaging, The Netherlands, http://svi.nl). To analyze the distribution of Foxj1 in nuclei, we segmented nuclei using the StarDist software 70, using the "2D_versatile_fluo" model provided by the authors (doi:10.1007/978-3-030-00934-2_30). For the beta-tubulin IV and cytokeratin-5 datasets, statistical analyses were done in Python 3.7, using the Matplotlib 3.4.2 api for the plots and scipy 1.6.3 for the statistical tests. For the FoxJ1 datasets, statistical analyses were done in the Graphpad Prism software v8.4.3.<br><br>For the analysis of ZO-1 distribution, the Zellige script was first used to extract the surface of the epithelium in each z-stack (script by C.T. and R.E.). Perturbations in epithelial packing were then measured using the TissueMiner toolkit (R.E., doi:10.7554/eLife.14334)<br>For mucociliary clearance analysis, bead tracks were extracted with the FIDJI package of ImageJ v2.1.0, using the Trackmate Fidji plugin v5.2.0. Statistical analyses were performed withe the GraphPad Prism v8.4.3 software.<br><br>The image analysis scripts used for computing beta-tubulin IV, cytokeratin 5, and Foxj1 distributions are available from S.R. upon reasonable request. The Zellige script used to extract the surface of the epithelium in ZO-1 labeled samples is available from R.E. upon reasonable request. The TissueMiner toolkit used to analyze perturbations in epithelial packing is available at: GitHub https://github.com/mpicbg-scicomp/tissue_miner#about

The Tissue Miner toolkit is referenced in the following publication: Etournay et al., Elife 2016, DOI 10.7554/eLife.14334

For manuscripts utilizing custom algorithms or software that are central to the research but not yet described in published literature, software must be made available to editors and reviewers. We strongly encourage code deposition in a community repository (e.g. GitHub). See the Nature Research guidelines for submitting code & software for further information.

Data

Policy information about availability of data

All manuscripts must include a data availability statement. This statement should provide the following information, where applicable:
- Accession codes, unique identifiers, or web links for publicly available datasets
- A list of figures that have associated raw data
- A description of any restrictions on data availability

Data supporting the findings of the present study are provided in the article and Supplementary Information files or from the corresponding authors upon reasonable request. Source data are provided with this paper.

Field-specific reporting

Please select the one below that is the best fit for your research. If you are not sure, read the appropriate sections before making your selection.

[x] Life sciences [ ] Behavioural & social sciences [ ] Ecological, evolutionary & environmental sciences

For a reference copy of the document with all sections, see nature.com/documents/nr-reporting-summary-flat.pdf

Life sciences study design

All studies must disclose on these points even when the disclosure is negative.

Sample size	Given the exploratory nature of the study, we did not perform statistical analyses to predetermine sample size. Instead we used 3 biological replicates to document infection by qPCR and immunofluorescence, as this sample size is considered appropriate to detect biologically relevant differences with these techniques.
Data exclusions	Data were excluded when epithelial cultures showed microbial contamination and when the porous membrane supporting the cultures appeared pierced upon microscopic examination.
Replication	Biological replicates represent epithelial cultures from cells of different donors. Experiments were performed on at least 3 biological replicates unless specified in the figure legend. All attempts at replication were successful. All antibodies used were from the same clone throughout the study and different lots were titrated to give similar fluorescence intensities.
Randomization	Randomization was not appropriate for this study as, for each biological replicate, control epithelial samples and infected epithelial samples were derived from the same donor. Hamsters were randomly assigned to the SARS-CoV-2 and Mock-infected groups.
Blinding	Blinding was implemented when experiments involved investigators in more than one group. Specifically, investigators using automated image analysis pipelines with standardized parameters were blinded to the group assignments. Setup experiments performed to validate the viral qPCR assay, the TCID50 assay, and the cytokine measurements were also performed in a blinded fashion, with one investigator doing the infections and another investigator who was blinded to the treatment doing the measurements.

Reporting for specific materials, systems and methods

We require information from authors about some types of materials, experimental systems and methods used in many studies. Here, indicate whether each material, system or method listed is relevant to your study. If you are not sure if a list item applies to your research, read the appropriate section before selecting a response.

Materials & experimental systems

n/a	Involved in the study	
[ ]	[x]	Antibodies
[ ]	[x]	Eukaryotic cell lines
[x]	[ ]	Palaeontology and archaeology
[ ]	[x]	Animals and other organisms
[x]	[ ]	Human research participants
[x]	[ ]	Clinical data
[x]	[ ]	Dual use research of concern

Methods

n/a	Involved in the study	
[x]	[ ]	ChIP-seq
[x]	[ ]	Flow cytometry
[x]	[ ]	MRI-based neuroimaging

Antibodies

Antibodies used	For each antibody, the following items are listed: source species, antigen, fluorochrome, clone, reference, and supplier.<br>Rabbit, beta-tubulin IV, AF488, clone EPR16775, ref. ab204003, Abcam<br>Rabbit, beta-tubulin IV, AF647, clone EPR16775, ref. ab204034, Abcam<br>Rabbit, cytokeratin 5, AF647, clone EP1601Y, ref. ab193895, Abcam<br>Rabbit, MUC5AC, AF555, clone EPR16904, ref.ab218714, Abcam<br>Rabbit, ZO-1, AF555, clone 1-A12, ref. MA3-39100-A555, Invitrogen<br>Mouse, SARS-CoV-2 spike, unconjugated, clone 702, gift from N. Escriou (in house manufacturing)<br>Goat, Foxj1, unconjugated, polyclonal, ref. AF3619, R&D Systems<br>Goat, mouse IgG (H+L) cross-adsorbed, AF488, polyclonal, ref. A-11001, Invitrogen<br>Goat, mouse IgG (H+L) cross-adsorbed, AF555, polyclonal, ref. A-21422, Invitrogen
Validation	All antibodies were validated by their manufacturer. Commercial antibodies chosen were further validated by citations in the primary literature. Relevant citations:<br><br>Rabbit, beta-tubulin IV, clone EPR16775, ref. ab204003 and ab204034, Abcam: rabbit monoclonal antibody cited in:<br>DOI 10.1016/j.chom.2020.05.020<br>DOI 10.1371/journal.ppat.1006962<br>DOI 10.5607/en.2019.28.3.362<br><br>Rabbit, cytokeratin 5, clone EP1601Y, ref. ab193895, Abcam: rabbit monoclonal antibody cited in:<br>doi: 10.7554/eLife.31657<br>doi: 10.1016/j.celrep.2019.07.007<br>doi: 10.1038/s41598-020-69948-2<br><br>Rabbit, MUC5AC, A clone EPR16904, ref.ab218714, Abcam: rabbit monoclonal antibody cited in:<br>doi: 10.1038/s41598-019-56737-9<br>doi: 10.1080/21691401.2019.1579732<br>doi: 10.3892/ijmm.2019.4054<br><br>Rabbit, ZO-1, clone 1-A12, ref. MA3-39100-A555, Invitrogen: mouse monoclonal antibody cited in 530 references including:<br>doi: 10.1242/jcs.208736<br>doi: 10.1038/ncomms10420<br>doi: 10.1038/nprot.2015.021<br><br>Goat, Foxj1, unconjugated, polyclonal, ref. AF3619, R&D Systems: goat polyclonal antibody cited in:<br>doi: 10.1038/s41598-019-52208-3<br>doi: 10.1371/journal.pone.0160216<br>doi: 10.1165/rcmb.2008-0073OC<br><br>The anti-SARS-CoV-2 spike mouse antibody (clone 702) was generated in house by the team of N. Escriou at Institut Pasteur. This antibody was validated by comparing its staining pattern in infected and uninfected samples of Vero-E6 cells, and in SARS-CoV-2 spike-expressing and non-expressing 293-T cells.

Eukaryotic cell lines

Policy information about cell lines

Cell line source(s)	SARS-CoV-2 viral supernatant titration was performed on Vero-E6 cells which were initially obtained from the ATCC. Reconstructed bronchial epithelia (MucilAirTM) were generated from human bronchial tissue at the Epithelix company (www.epithelix.com).
Authentication	The Vero-E6 cell line was not authenticated.
Mycoplasma contamination	Vero-E6 cells were routinely tested for mycoplasma contamination and were found negative.
Commonly misidentified lines (See ICLAC register)	No commonly misidentified cell lines were used in this study.

Animals and other organisms

Policy information about studies involving animals; ARRIVE guidelines recommended for reporting animal research

Laboratory animals	Male Syrian hamsters (Mesocricetus auratus) of 5-6 weeks of age (average weight 60-80 grams) were purchased from Janvier Laboratories and handled under specific pathogen-free conditions.

Wild animals	The study did not involve wild animals.
Field-collected samples	The study did not involve samples collected from the field.
Ethics oversight	Hamsters were handled according to the French legislation and to the regulations of Pasteur Institute Animal Care Committees, in compliance with the European Communities Council Directives (2010/63/UE, French Law 2013–118, February 6, 2013). The Animal Experimentation Ethics Committee (CETEA 89) of the Pasteur Institute approved this study (200023; APAFIS#25326-2020050617114340 v2) before experiments were initiated. Hamsters were housed by groups of 4 animals, with ad libitum access to water and food. Animals were manipulated in class III safety cabinets in the Pasteur Institute animal facilities accredited by the French Ministry of Agriculture for performing experiments on live rodents. Before any manipulation, animals underwent an acclimation period of one week. All animals were handled in strict accordance with good animal practice.

Note that full information on the approval of the study protocol must also be provided in the manuscript.
